# Differential expression of lung adenocarcinoma transcriptome with signature of tobacco exposure

**DOI:** 10.1007/s13353-020-00569-1

**Published:** 2020-06-20

**Authors:** Raneem Y. Hammouz, Joanna K. Kostanek, Aleksandra Dudzisz, Piotr Witas, Magdalena Orzechowska, Andrzej K. Bednarek

**Affiliations:** Department of Molecular Carcinogenesis, Medical University of Lodz, Zeligowskiego 7/9, 90-752 Lodz, Poland

**Keywords:** Lung adenocarcinoma, Tobacco, Non-smokers, Differentially expressed genes, Sex disparity, EMT

## Abstract

Smoking accounts for almost 80–90% of lung cancer cases, which is also the most frequent cause of cancer-related deaths in humans. With over 60 carcinogens in tobacco smoke, cells dividing at the time of carcinogen exposure are at particular risk of neoplasia. The present study aimed to investigate global gene expression differences in lung adenocarcinoma (LUAD) tumour samples of current smokers and non-smokers, in an attempt to elucidate biological mechanisms underlying divergent smoking effects. Current and non-smoker tumour samples were analysed using bioinformatics tools, examining differences in molecular drivers of cancer initiation and progression, as well as evaluating the effect of smoking and sex on epithelial mesenchymal transition (EMT). As a result, we identified 1150 differentially expressed genes showing visible differences in the expression profiles between the smoking subgroups. The genes were primarily involved in cell cycle, DNA replication, DNA repair, VEGF, GnRH, ErbB and T cell receptor signalling pathways. Our results show that smoking clearly affected E2F transcriptional activity and DNA repair pathways including mismatch repair, base excision repair and homologous recombination. We observed that sex could modify the effects of *PLA2G2A* and *PRG4* in LUAD tumour samples, whereas sex and smoking status might possibly have a biological effect on the EMT-related genes: *HEY2*, *OLFM1*, *SFRP1* and *STRAP*. We also identified potential epigenetic changes smoking solely might have on EMT-related genes, which may serve as potential diagnostic and prognostic biomarkers for LUAD patients.

**Electronic supplementary material:**

The online version of this article (10.1007/s13353-020-00569-1) contains supplementary material, which is available to authorized users.

## Background

Lung cancer represents 12.7% of all new cancer cases and is also the most frequent cause of cancer-related deaths worldwide accounting for 18.2% (Ferlay et al. 2010). Tobacco smoke exposure accounts for the majority of lung cancer cases; however, almost 25% of worldwide cases occur in lifelong never smokers (Lee et al. 2011). Never smokers (NS) are defined as individuals who have smoked less than 100 cigarettes during their lifetime (Couraud et al. 2015). Consequently carcinogens in cigarette smoke are presumed to induce carcinogenesis in smokers; however, no such affront exists in NS implying an alternative mechanism. Thus, lung cancer in lifelong never smokers (LCINS) is considered a separate entity with adenocarcinoma predominance (Yano et al. 2008), over squamous cell carcinoma (SCC) (Bhopal et al. 2019). The UK Million Women Study found three significant risk factors out of 34 potential ones, to be associated with an increased incidence of LCINS and included non-white ethnicity, asthma requiring treatment and tall stature (Pirie et al. 2016).

Major contributors to lung cancer include secondhand tobacco smoking, especially with the changes in cigarette manufacturing practices and smoking habits allowing deeper aspiration (Gridelli et al. 2015), occupational carcinogen exposure, pollution, X-ray radiation and radon exposure (Field and Withers 2012). In populations with lung cancer, the 5-year survival varies from 4 to 17% depending on the stage and regional differences (Hirsch et al. 2017). Three studies have supported the finding that smoking duration rather than intensity serves as a predictor for lung cancer and the differences between the histological types. Additionally, the association of lung cancer with tobacco smoking differs by histological type according to the exposure of the respiratory tract site to tobacco smoke particles, with lower exposure to the peripheral and subpleural regions. In small cell and squamous cell carcinoma, a rapid increase in risk was observed with the number of cigarettes smoked following smoking cessation, whereas to a lesser extent in adenocarcinoma (Kenfield 2008; Doll and Peto 1978; Flanders et al. 2003). Nicotine-mediated tumour promotion is made possible through the activation of the α7 subunit of the nicotine acetylcholine receptors (α7-nAChR) or β-adrenergic receptors activating the signalling cascade. In NSCLC, the expression of (α7-nAChR) is higher in squamous carcinoma smokers than in adenocarcinoma (Pezzuto et al. 2019).

In NSCLC, local progression and metastasis has been associated with epithelial to mesenchymal transition (EMT). In adenocarcinoma and SCC, there is a strong correlation between the expression of EMT biomarkers and an advanced stage and poor differentiation (Mahmood et al. 2017). Little is known regarding the epigenetic changes of the molecular mechanisms involved in EMT in NSCLC. Cigarette smoking plays a vital role in promoting EMT and is associated with poor survival, cell migration and invasion in NSCLC through the deregulation of E-cadherin. Cigarette smoke condensation (CSC) induces EMT by downregulating E-cadherins, which are considered tumour suppressors and important molecular markers in smoking patients (Nagathihalli et al. 2012).

Robust sex differences in terms of higher incidence of DNA damage in men compared to women exist with a male cancer incidence predominance (Dorak and Karpuzoglu 2012). While these differences might be mediated through acute sex hormonal action paralleling the variation in circulating sex hormone concentrations, factors independent from acute hormone action underlie this discrepancy. However, there is insufficient data to make a clear determination regarding the impact of sex, primarily due to the fact that sex differences have not been exclusively analysed. With LCINS being the seventh most prevalent in the world, a higher proportion also seems to be amongst women in comparison to men (Bhopal et al. 2019). Oestrogen receptor α and β are more frequently expressed in lung tissue compared to normal tissue, whereas progesterone receptors are less frequently expressed in cancerous tissue, with the receptor’s expression profile possibly correlating with the outcome (Couraud et al. 2012). In brain tumour glioblastoma, sex differences play a role in patient outcomes beyond hormonal influences, appearing to be intrinsic to the tumour cells. Yang et al. proposed that patient sex correlates with prognosis as well as treatment (Yang et al. 2019).

Recent work identifying further mechanisms of acquired resistance in NSCLC necessitates additional detailed understanding of the biological underpinnings of this tumour phenotype. An improved understanding of LUAD tumour biology accounting for smoking status and sex will allow a better understanding of available well-known modulators of treatment response and toxicity. FDA has been recommending systematic analysis of sex differences that will allow precision oncology rather than just exploring molecular markers. This paper presents a few major genetic changes occurring in current and non-smokers LUAD tumour samples and examining the effect of smoking on both sexes and EMT. Our approach to a pathway-specific gene association analysis will help form a better view of the accumulative effect of group functionally related genes aiding in revealing the transcriptional program accounting for the variability in cancer phenotype.

## Materials and methods

### Data collection

The expression profile data of lung adenocarcinoma level 3 normalised (illuminahiseq_rnaseq) including 522 samples with their corresponding clinical information were manually retrieved from The Cancer Genome Atlas (TCGA) database (http://gdac.broadinstitute.org/ data status of Jan 28, 2016). For validation, the gene chip GSE10072 of LUAD with its clinical manifestation data was also downloaded from the Genome Expression Omnibus (GEO) database for 180 patients (https://www.ncbi.nlm.nih.gov/geo/). Raw data has been processed, and patients with missing clinical/expression values were excluded from the analysis. Finally, the study included 146 patients from TCGA and 45 probes from GEO databases. Further grouping according to smoking status included a permissible value of 1 for lifelong non-smokers (NS) and 2 for current smokers (CS). We excluded reformed smokers to adjust for smoking because residual confounding could lead to some of the excess hazards amongst the groups. Our study included 54 NS of which 13 were males and 41 females and 92 CS of which 59 and 33 were male and female LUAD patients, respectively. A series of clinical traits for TCGA patients are shown in Supplementary Table S1.

### In silico analysis

To further explore the optimal molecular subgroups in LUAD, unsupervised hierarchical clustering was performed on the patient tumour samples (T), with Pearson correlation and the pairwise complete-linkage method, separately on the subgroups (NS and CS). ExpressCluster software (CBDM Bioinformatics, Scott Davis n.d) was used to find common and unique expression profiles of genes showing differentiation between the patient T subgroups. Profiles indicating contrast between CS and NS subgroups were considered significant. Clustering was also performed by applying K-means++ algorithm, Z-norm signal transformation and rank correlation distance metric. *p* values were corrected using Benjamini and Hochberg adjustment to minimise errors, and adjusted *p* values < 0.05 were considered significant. Profiles indicating contrast between CS and NS were considered significant. To investigate the molecular differences amongst the groups, associated clinical manifestations including history of smoking, treatment response, sex, age, stage and vital status were analysed.

### Multivariate and principal component analysis

Multivariate analysis (MFA) was used to estimate the relationships amongst gene expression patient subgroup correlates, in order to identify partitioning of patient subgroups. MFA standardises variables in predefined blocks of data calculating the global axes, which are the linear combination of the original parameters, thus maximising the global data variance. MFA was computed using the R packages FactoMineR and factoextra (Lê et al. 2008). Principal component analysis (PCA) was used to reduce the dimensionality of the subsets, creating uncorrelated variables and maximising variance (Jolliffe and Cadima 2016) between subgroups.

### Gene set enrichment analysis

Gene set enrichment analysis (GSEA) (Subramanian et al. 2005) was carried out to determine whether the identified set of genes showed significant molecular functions between the patient subgroups. Datasets and phenotype label files were created (defined either as CS or NS and males or females) and loaded onto the GSEA software (v3.0). Enrichment analysis was performed according to the expression data for NS and CS, separately, to all 20,501 genes in terms of canonical pathway GeneSet (CP), cellular components (CC), molecular function (MF) and transcription factor targets (TFT) obtained from the Molecular Signatures Database (MSigDB). The number of permutations was set to 1000, and a ranked list metric was generated using *t* test yielding the functions involved in both the high and low expression groups. The nominal *p* value of enrichment results cut-off was 0.05, while gene clusters with false discovery rate, FDR < 0.25, were considered statistically significant. FDR < 0.25 was chosen rather than 0.05 as according to GSEA, an FDR < 0.25 is likely to generate interesting hypotheses for further research (Subramanian et al. 2005). Random seed for permutation to achieve repeatability was 779,948,241, with the parameter of permutations set as a 1000.

### Weighted gene correlation network analysis

WGCNA R package was used to construct a scale-free network from gene expression data (Langfelder and Horvath 2008). Its algorithm was used to screen out the power value in the construction of modules. The appropriate power (9) was used following the use of the gradient method testing the independence and the average connectivity degree of different modules. We made sure to select the power when the degree of independence *R*^2^ was greater than 0.8 (*R*^2^ = 0.94 and slope = − 1.48). We then proceeded to construct the modules with minModuleSize set as 30 to achieve high reliability of the results. Following that, the correlation was transformed into adjacency matrix then into a topological overlap measure (TOMplot). Heatmap tools package (Babicki et al. 2016) was used to analyse the strength of the interactions. Module-trait associations were estimated using the correlation between the module eigengene and the smoking status (clinical trait), which allowed identification of module (expression set) highly correlated to the phenotype. Gene significance (GS) for each expression profile was calculated as the absolute value of the correlation between the expression profile and the trait; module membership (MM) was defined as the correlation of expression profile and each module eigengene.

### Functional enrichment analysis of the co-expression modules

The constructed modules had different numbers of genes, and functional enrichment analysis was performed on the significantly distinct modules. Gene ontology (GO) analysis results from GOenr (enrichmentAnalysis) in WGCNA were extracted out. *p* value ≤ 0.05 after correction was used as a threshold. GO terms that were significantly overrepresented in a set of genes were then visualised in Cytoscape 3.5 (Shannon 2003), to further explore functions and pathways related to DEGs in the subgroups. All metabolic pathways generated were further subjected to enrichment and topological analysis using Java Enrichment of Pathways Extended to Topology (JEPETTO) plugin, and co-expressed modules were constructed by the Cytoscape software to identify hub genes. JEPETTO allows identifying novel and significant relationships using protein-protein interaction networks based on KEGG database (Winterhalter et al. 2014). It helped us analyse the functional association of the top-ranked underlying networks between DEGs and known cellular pathways. ViSEAGO R package (Brionne et al. 2019) was also used for biological interpretations of biological processes (BP) category using the GO public database. Associated gene terms were retrieved from EntrezGene database (Nov 2019) for Homosapiens ID 9606, and experimental GO terms from orthologous genes were added. Enrichment tests were performed using Fisher test, subsequently with classic, elim, weight and weight01 algorithms. We used several algorithms to validate the robustness of enrichment. Enriched GO terms (*p* < 0.01) were grouped into functional clusters using hierarchical clustering with the help of GOSemSim (Yu et al. 2010) based on Wang’s semantic similarity between GO terms respecting GO graph topology and Ward’s criterion. Further, these functional clusters were grouped using hierarchical clustering based on BMA distance between sets of GO terms and Ward’s criterion.

### Statistical analysis

Mutation and the frequency of copy number alterations (CNA) were analysed for association between smoking groups. Mutation and CNA were downloaded from cbioportal website (https://www.cbioportal.org/) for lung adenocarcinoma (TCGA, Firehose Legacy); data was merged according to the patient ID. The Pearson’s chi-squared test was used to compare the mutation and CNA frequency between the subgroups. A Bonferroni-corrected *p* value of < 0.05 was used to determine the significance threshold for all association tests. Differences in the EMT marker gene expression signatures according to patient category (both sex and smoking) were examined using the Kruskal-Wallis analysis of variance, followed by the Dunn post hoc with Bonferroni correction for multiple comparisons. *p* < 0.05 was considered statistically significant. Computation was performed using the dunn.test R package, as well as GraphPad 5.01 (GraphPad Prism7 Software n.d, CA, USA).

## Results

### Unsupervised cluster analysis identifies six subgroups for LUAD current and non-smoker patients

Unsupervised hierarchical clustering analyses of 120 CS and 75 NS tumour samples were conducted with 20,501 genes downloaded from TCGA. These lung tumour samples were then divided into six subgroups, three each, based on molecular signature differences as shown in Supplementary Fig. S1. CS subgroup 1 (C1) contained 54 samples, subgroup 2 (C2) contained 38 samples and subgroup 3 (C3) contained 28 samples, whereas for NS, subgroup 1 (N1) contained 17 samples, subgroup 2 (N2) 37 samples and subgroup 3 (N3) 21 samples.

### Classification of the patient subgroups and identification of DEGs

Gene set enrichment analysis (GSEA) allowed identification of genes contributing to the differentiation of the smoking status of the subgroup tumour samples. The 100 best genes (obtained from GSEA heatmaps) differentiating the compared CS vs NS subgroups were extracted following 9 different group combinations (Supplementary Fig. S2). PCA was used to explore the 6 patient subgroups, assessing the variation across each category (CS or NS) according to smoking status (Supplementary Fig. S3). Subgroups C1, C2 combined verses N1 and N2 also combined were mutually independent and highly clustered in intra-subgroups, thus dividing them based on their molecular differences. Subgroups N3 and C3 were eliminated from further analysis, rendering four subgroups best suitable to discriminate LUAD tumour samples according to their smoking status (Supplementary Fig. S4). We then identified from ExpressCluster 12 clusters showing unique expression profiles differentiating C1, C2, N1 and N2 subgroups yielding 2454 DEGs. Multivariate analysis of 2454 gene expression revealed clear distinction between C1, C2, N1 and N2 (Supplementary Fig. S5).

### Gene co-expression network reveals 1150 DEGs corresponding to smoking status

Expression values of the 2454 genes were used to construct the co-expression modules by WGCNA package tool. To identify modules of these genes, the topological overlap-based dissimilarity was determined and used as a distance measure with a constant height cut-off value (6 × 10^5^) (Supplementary Fig. S6). Soft threshold power was set to 9 in which *R*^2^ was 0.94 to ensure a scale-free network (Supplementary Fig. S7). This resulted in merging of highly correlated eigengenes, and the 9 best modules differentiating between CS and NS were chosen (Supplementary Figs. S8 and S9). We then combined the module genes and removed duplicates generating 1150, permitting the identification of distinct transcription modules for subgroups (CS and NS) and considered as our candidate genes.

### Functional annotation analysis of DEGs

To gain insights into the biological functions that may be affected due to tobacco exposure, we performed functional analysis based on gene ontology (GO) using GSEA, JEPETTO plugin in Cytoscape and ViSEAGO package in R. GSEA enrichment of gene sets according to GO functional terms associated with cellular process led to the identification of processes mostly relating to ECM regulation and replication fork (Supplementary Table 2 and Supplementary Fig. 8), while cell cycle, DNA repair, DNA replication and proliferation and E2F pathway and transcription family targets for canonical pathways (Supplementary Tables 3 and 4 and Supplementary Fig. 10). The biological process controlled by the genes in the “turquoise” module was found to be mainly enriched in cell cycle regulation, division, DNA replication, repair and E2F pathway, while “grey” module genes were associated with proliferation, transcription, cell to cell signalling and P53 downstream signalling pathway and other modules such as “magenta” with cytoskeletal organisation, “dark orange” with skeletal development and “black” with immune responses (Supplementary File 1). Pathway analysis using JEPETTO plugin in Cytoscape revealed that cell cycle, DNA replication and repair pathways (MMR, BER, NER), HR, ErbB GnRH, TGF-β, T cell receptor and VEGF signalling pathway as major pathways varying with different constitution for CS and NS analyses and asthma (Table 1). However, when analysed separately, overexpressed genes in CS seem to correlate with mismatch repair, cell cycle, NHEJ and ABC transporters, while in NS, only asthma seemed to be significantly enriched (Supplementary Table 5).

**Table 1:** Genes involved in major cancer-related pathways coloured according to increased expression, CS in red and NS in blue with their corresponding NCBI Reference Sequence accession number

Cell cycle	*CD22*, *BTK*, ***PIK3R5***(NG_030374.1), ***PIK3CG*** (NG_050579.1), *CD19* (NG_007275.1)*, PRKCB* (NG_029003.2)*, INPP5D* (NG_033988.1)*, NFATC2, PIK3R1*(NG_012849.2)*,PIK3AP1,FCGR2B*(NG_023318.1)*, RAC2* (NG_007288.1)
Mismatch repair	***POLD1*** (NG_033800.1)*,****RFC5, RFC4, RFC3,****EXO1* (NG_029100.2)
Homologous recombination	***POLD1*** (NG_033800.1), *RAD54B* (NG_012878.2), *XRCC2* (NG_027988.2), *EME1* (NG_029665.1), *RAD51* (NG_012120.1), *RAD54L* (NG_012144.1), *BLM* (NG_007272.1)
DNA replication	*DNA2* (NG_034247.1)*, MCM2* (NG_050771.1)*,****POLD1*** (NG_033800.1)*, POLE2* (NG_052877.1)*, POLE* (NG_033840.1)*,****RFC3, RFC4, RFC5***
VEGF signalling pathway	***PIK3R5*** (NG_030374.1)***, PLA2G2A*** (NG_012928.1), ***PIK3CG*** (NG_050579.1), ***PRKCB*** (NG_029003.2), ***NFATC2 PLA2G5*** (NG_032045.1), ***PIK3R1*** (NG_012849.2), ***PLA2G2D, RAC2*** (NG_007288.1)
T cell receptor signalling pathway	***CD40LG*** (NG_007280.1), *CSF2* (NG_033024.1), *CD28* (NG_029618.1), *PTPRC* (NG_007730.1), ***PIK3R5*** (NG_030374.1), *CD4* (NG_027688.1), *ITK* (NG_016276.1) ***PIK3CG*** (NG_050579.1)***, NFATC2, PIK3R1*** (NG_012849.2)
ErbB signalling pathway	***PIK3R5*** (NG_030374.1), ***PIK3CG*** (NG_050579.1), ***MAPK10*** (NG_013325.2), ***PRKCB*** (NG_029003.2), ***PIK3R1*** (NG_012849.2), *BTC*
GnRH signalling pathway	***PLA2G2A*** (NG_012928.1), ***MAPK10*** (NG_013325.2), *PLCB2* (NG_052867.1), *CACNA1C* (NG_008801.2), ***PRKCB*** (NG_029003.2), *PTK2B* (NG_029510.2), ***PLA2G5*** (NG_032045.1), ***PLA2G2D***
Base excision repair	*TDG*
Nucleotide excision repair	***POLD1*** (NG_033800.1), *POLE2* (NG_052877.1)*,* *POLE* (NG_033840.1), ***RFC3****,****RFC4****,****RFC5***
TGF beta signalling pathway	*TGFBR2 (*NG_007490.1)
Asthma	***CD40LG*** (NG_007280.1)

### Smoking dysregulates cell cycle

Of our 1150 genes JEPETTO analysis for cell cycle related genes (*CD22*, *BTK*, *PIK3R5*, *PIK3CG*, *CD19*, *PRKCB*, *INPP5D*, *NFATC2*, *PIK3R1*, *PIK3AP1*, *FCGR2B* and *RAC2*) showed that their expression was elevated in NS compared to CS. The expression levels of these genes *PIK3R5*, *PIK3CG*, *PIK3R1* NFATC2 and *RAC2* in the NS cohort also seemed to be elevated in mismatch repair, homologous recombination, DNA replication, VEGF signalling pathway, T cell receptor signalling pathway, ErbB signalling pathway and GnRH signalling pathway (Table 1). GSEA analysis of the 1150 genes brought up **REACTOME_E2F_MEDIATED_REGULATION_OF_DNA_REPLICATION** (Table 2).

**Table 2:** Reactome pathways from functional enrichment STRING for 1150 DEGs, cell cycle and E2f related genes (CS in red, NS in blue)

Reactome pathways	FDR value	Genes from our DEGs
Activation of E2F1 target genes at G1/S (9 of 28 genes)	0.0003	*CDK1, RRM2, TK1, TYMS, CDC6, CDC45, ORC1, CDT1, E2F1*
E2F-enabled inhibition of pre-replication complex formation (5 of 9 genes)	0.0029	*CCNB1, CDK1, ORC1, ORC6, MCM8*
Transcriptional regulation by E2F6 (6 of 32 genes)	0.0412	*RRM2, E2F1, CDC7, CHEK1, BRCA1, EZH2*

**Fig. 1:**
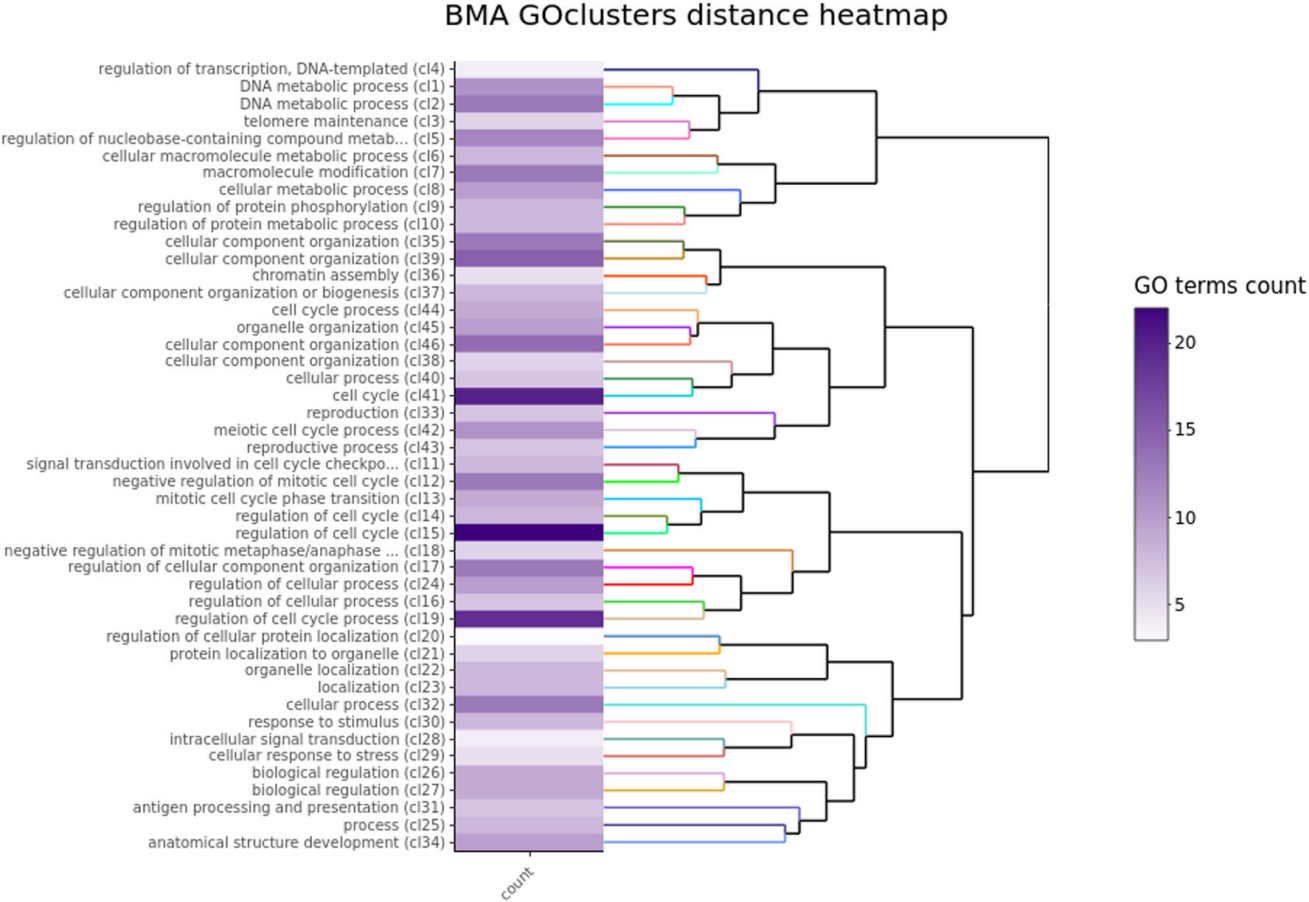
Functional analysis of 113 genes. The clustering heatmap plot of functional sets of gene ontology (GO) terms was obtained using ViSEAGO showing the major biological processes, cluster number and number of genes in each cluster. Based on BMA semantic similarity distance and Ward’s clustering criterion

We also found 113 genes relating to cell cycle (Table 3) to be overexpressed in our CS cohort; 28 were identified with a log fold change over 1.5. Further GO analysis of the 113 genes using ViSEAGO R tool returned 46 clusters, relating mostly in to DNA replication, cell cycle checkpoint, regulation and response to chemotherapy drugs (Fig. 1), in particular GO:0051726, regulation of cell cycle; GO:0010564, regulation of cell cycle process; GO:0051726, regulation of cell cycle; and GO:1901987, regulation of cell cycle phase transition (Supplementary File 2). Additionally, *BLM* and *RAD51* were statistically significant and enriched with overexpression in CS for GO:1901563, response to camptothecin; GO:0097329, response to antimetabolite; and GO:0072710, response to hydroxyurea (Supplementary File 2) (Supplementary Table 14)**.**

**Table 3:** 113 cell cycle-related genes with log fold change values LogFC < 1.5

GENE	AVG CS	AVG NS	CS/NS	LOG2FC
ATAD2	1473.042	733.996	2.007	1.005
**AURKB**	412.571	138.189	2.986	*1.578*
**BUB1**	739.387	287.960	2.568	*1.360*
CCNB1	1245.186	577.687	2.155	1.108
**CDC45**	325.860	120.739	2.699	*1.432*
CDC6	674.595	324.381	2.080	1.056
CDC7	307.452	170.302	1.805	0.852
**CDCA2**	170.324	65.480	2.601	*1.379*
CDK1	1082.311	492.400	2.198	1.136
**CDT1**	534.413	210.613	2.537	*1.343*
**DLGAP5**	496.263	197.427	2.514	*1.330*
E2F1	692.107	327.445	2.114	1.080
E2F8	237.507	112.652	2.108	1.076
EZH2	533.852	287.415	1.857	0.893
FAM83D	491.591	217.409	2.261	1.177
MAD2L1	553.410	263.098	2.103	1.073
MCM8	386.794	211.137	1.832	0.873
TK1	1630.038	851.468	1.914	0.937
TYMS	882.919	518.395	1.703	0.768
RRM2	1789.905	720.811	2.483	*1.312*
CHEK1	358.058	153.272	2.336	1.224
BRCA1	463.460	258.389	1.794	0.843
**MELK**	464.370	184.240	2.520	*1.334*

### Analysis of multiple omics data

Of the 1150 DEGs, we identified 130 genes with log fold change − 1.5 < log_2_FC < 1.5 and reduced them to 25 genes with a log fold change of − 2 < log_2_FC < 2 (Supplementary Table 6), indicating distinct molecular signatures between CS and NS LUAD patients with biological differences amid them between (Table 4). The log2 ratio is interpreted as the average log fold change in gene expression between the smoker groups. Fifteen genes seem to be overexpressed in NS and 10 in CS. Enrichment analysis of those 25 genes did not bring up significant metabolic pathways, but a few of them seem to be reported in the literature as tumour suppressors or involved in EMT process. *SCGB1A1* seems to be an important defence molecule of the lung and one of the top hypermethylated genes in smokers. *PRH2* has not been reported in the literature regarding any inflammatory diseases, however showed a log_2_FC of 2.76 in CS.

**Table 4:** 1150 DEGs with log fold change values − 2 < LogFC < 2

Name	Average CS	Average NS	CS/NS	LogFC
*PRG4*	76.328	4765.678	0.016	− 5.964
*OGN*	52.414	523.019	0.100	− 3.319
*SCGB1A1*	841.129	5499.275	0.153	− 2.709
*C19orf59*	89.226	501.220	0.178	− 2.490
*ADH1B*	461.322	2309.951	0.200	− 2.324
*ADAMTS8*	38.365	187.606	0.204	− 2.290
*FCER2*	8.227	40.048	0.205	− 2.283
*RETN*	18.094	84.525	0.214	− 2.224
*AGER*	746.866	3459.086	0.216	− 2.211
*C10orf105*	6.680	30.612	0.218	− 2.196
*HSD17B6*	240.568	1098.694	0.219	− 2.191
*C6*	35.335	146.531	0.241	− 2.052
*ABCA8*	77.462	315.615	0.245	− 2.027
*INMT*	284.509	1141.019	0.249	− 2.004
*C1QTNF7*	35.305	141.375	0.250	− 2.002
*HES6*	1302.454	314.761	4.138	2.049
*GPC2*	88.922	21.271	4.181	2.064
*GCLC*	3866.484	922.099	4.193	2.068
*LRRC16B*	59.894	14.186	4.222	2.078
*MLLT11*	652.937	147.855	4.416	2.143
*GLDC*	230.230	50.770	4.535	2.181
*HPDL*	80.714	15.912	5.073	2.343
*MTL5*	171.734	33.037	5.198	2.378
*PRH2*	34.155	5.375	6.354	2.668
*ABCC2*	645.006	43.092	14.968	3.904

### Characteristics of LUAD patients related to different EMT profiles in association with smoking status

**Fig. 2:**
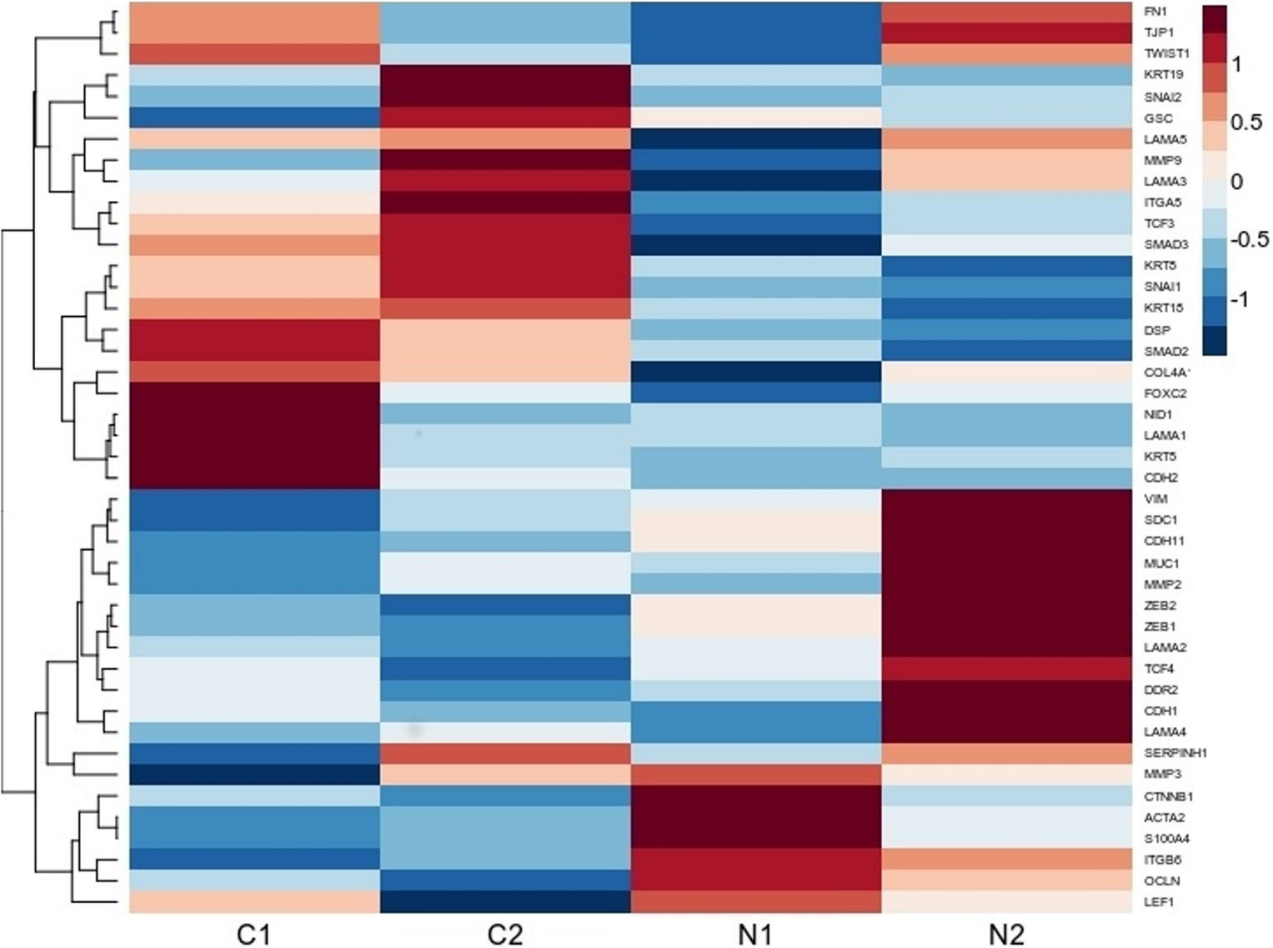
The heatmap for EMT markers in current and never smoker LUAD patients, showing contrasting profiles of expression between the smoking groups for some genes

We performed dimensional grouping through MFA of LUAD patients according to the smoking status of the different subgroups. Our results show a contradictory pattern for EMT members between NS and CS. *ITGA5*, *TCF3*, *SMAD3*, *KRT5*, *SNAI1*, *KRT18*, *DSP* and *SMAD2* are overexpressed in CS and underexpressed in NS, whereas *VIM*, *SDC1*, *CDH11*, *MUC1*, *MMP2*, *ZEB2*, *ZEB1*, *LAMA2*, *TCF4*, *DDR2*, *CDH1*, *LAMA4*, *MMP3 ITGB6* and *OCLN* are underexpressed in CS and overexpressed in NS (Fig. 2). Kruskal-Wallis analysis on all 43 EMT genes identified 25 to be statistically significant. None showed significance across all 6 group combinations (C1C2, C1N1, C2N1, C1N2, C2N2, N1N2). However of the statistically significant EMT genes, the Dunn post hoc test for both smoking subgroups C1 and C2 showed significant difference in the expression of *KRT8* (*p* < 0.0108) and *TCF3* (*p* < 0.0134), while *LAMA2* (*p* < 0.0281), *LAMA3*(p < 0.010), *LAMA5* (*p* < 0.05) and *TJP1*(*p* < 0.0423) differed for both never smoker subgroups (N1, N2) (Supplementary Table 13).

### Gene expression analysis with special reference to EMT markers according to smoking status and sex

**Fig. 3:**
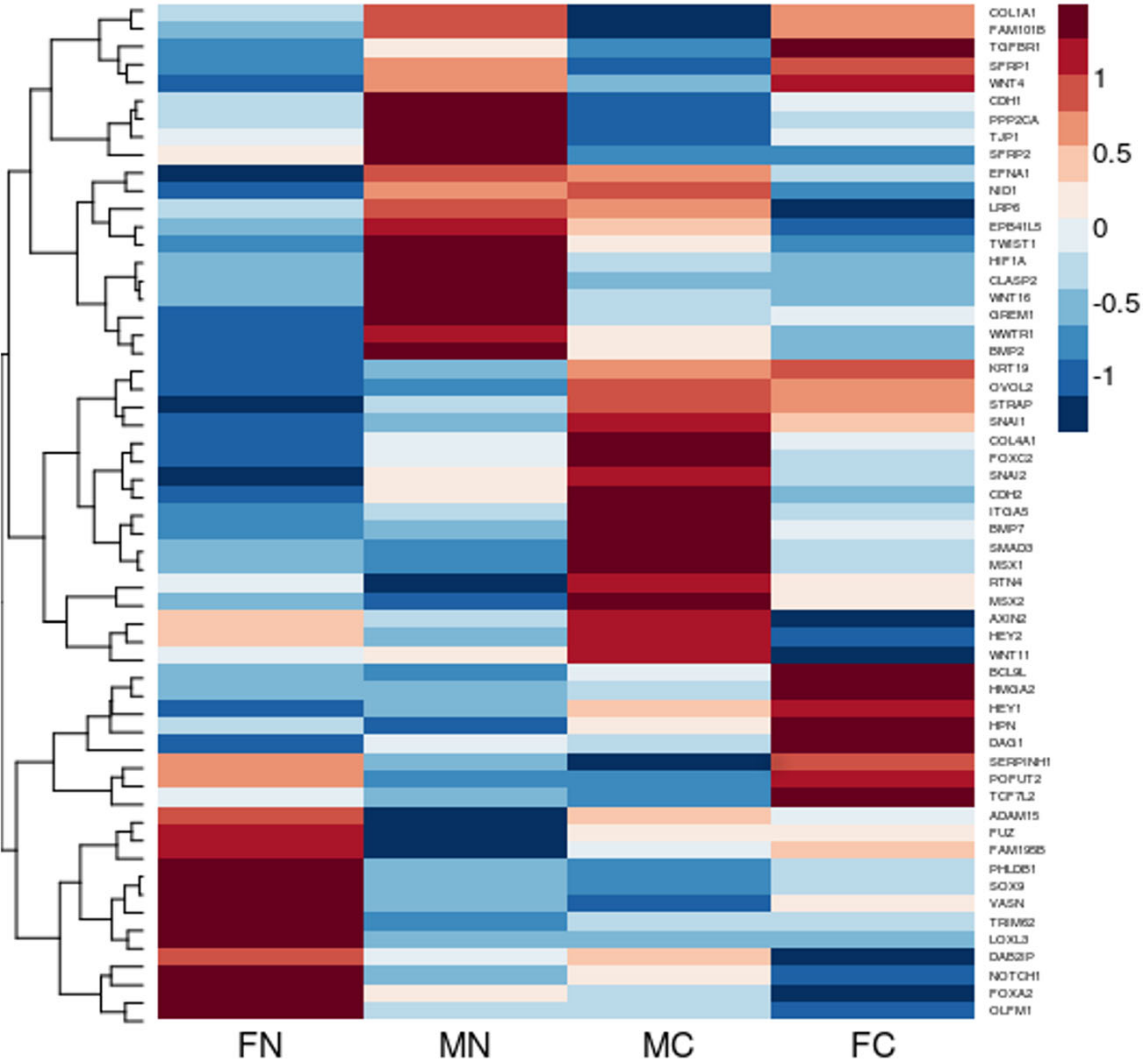
The heatmap for EMT marker genes showing contrasting profiles of expression in relation to smoking status and gender in our patient groups: female never smokers (FN), male never smokers (MN), female current smokers (FC) and male current smokers (MC)

Following analysis of 144 EMT markers, hierarchical clustering identified 57 genes with differing expression in relation to smoking status and gender in our patient groups: female never smokers (FN), male never smokers (MN), female current smokers (FC) and male current smokers (MC) (Fig. 3). We found 4 genes to be statistically significant between all four groups using Kruskal-Wallis and Dunn post hoc test (FN, FC, MN, MC): *OLFM1* (*p* < 0.0001), *HEY2* (*p* < 0.0378), *SFRP1* (*p* < 0.0109) and *STRAP* (*p* < 0.0001). *HEY2*, *OLFM1* and *STRAP* showed statistically significant difference between FN and FC groups, *OLFM1* and *STRAP* between groups FN and MC and, finally, *SFRP1* for MN and MC (Supplementary Table 7).

### Gender-related differences in relation to smoking status

We also performed GSEA enrichment analysis for male and female LUAD patients. In *males* of the significantly enriched datasets included “calcium mediated signalling”, “MYD88 dependent TLR receptor signalling pathway”, “Negative regulation of IL-6 production”, “Regulation of PI3K”, “Inositol phosphate biosynthetic process”, “Response to wounding”, “Respiratory burst”, “Sialyation”, “TLR4 signalling pathway” and “Positive regulation of stem cell differentiation”, whereas within **female** patients of the upregulated gene sets seemed to be focused around cell division, cell cycle regulation, nuclear division, chromatin remodelling and segregation and extracellular matrix organisation (Supplementary File 3). The log fold change between both sexes was also calculated, with *PLA2GA* and *PRG4 presenting with a* 1.5 < log_2_FC < − 1.5 in both NS and CS groups (Supplementary Table 8). Additionally DNA repair pathways BER, MMR and HR and DNA replication had a higher gene expression in males compared to females (Supplementary Table 9).

### Mutation analysis in different smoking subgroups

The total mutation count was found as expected to be higher in CS with a total of 10,471 in the 92 patients, when compared to a total of 1662 mutations in the 55 NS patients (data not included). We also found 7 significant gene mutation in all 4 subgroups *EGFR p* value = 0.002245, *LRP1B p* value = 0.006958, *ZFHX4 p* value = 0.006958, *MUC16 p* value = 0.007124, *RYR2 p* value = 0.02769, *NAV3 p* value = 0.03284 and *TP53 p* value = 0.04229 (Supplementary Table 10). *BRAF* mutation was also only found in CS with none at all in our NS cohort, in parallel with a few other studies (Paik et al. 2011). There are several other studies (Chapman et al. 2016; Midha et al. 2015; Dias et al. 2017; Takamochi et al. 2013) supporting our result with the higher incidence of *EGFR* mutation in NS groups compared to current/heavy smokers.

## Discussion

The goal of this study was to examine the consequences of tobacco smoking on lung adenocarcinoma (LUAD) transcriptome differentiation. Here we highlight that smoking promotes gene expression changes critical to tumour differentiation rather than initiation. Lung cancer is the global cause of death and 80–90% of these cases attributable to tobacco exposure. With over 60 carcinogens in cigarette smoke, the effects of tobacco on lung cancer genome are well established, making it a major influence on mutational burden driven by the exogenous carcinogens and endogenous DNA damage (Yoshida et al. 2020). Smoking lung cancer patients have the highest somatic alterations, approximately 10 times as many as those from non-smokers (Vogelstein et al. 2013), with G to T transversion being a molecular signature of tobacco smoke (TS) mutagens in lung cancer due to PAH exposure (Pfeifer et al. 2002). Additionally, TS increases driver mutations, cell-to-cell heterogeneity and other indirect effects such as inflammation, immune suppression and infection (Yoshida et al. 2020).

This is the first study, to our best of knowledge, to show systemic differences in gene expression of major processes involved in carcinogenesis including: cell cycle; DNA repair pathways (*MMR*, *HR*, *BER*, *NER*); DNA replication; and *ErbB*, *GnRH*, *VEGF*, *TGF-β* and T cell receptor signalling pathway between LUAD tumour samples in relation to smoking status. We also found previously reported asthma associated genes amongst our DEGs with elevated expression in non-smokers (NS). Asthma is suggested to increase lung cancer risk perhaps through damage caused by trauma or inflammation. Even non-smoking asthmatic patients seem to have an increased risk for lung cancer (Pirie et al. 2016; Qu et al. 2017). Damage caused by smoking amongst other factors affects DNA repair pathways and thus is linked to the genetic predisposition to cancer in several different tissues. For instance, inherited mutations in DNA mismatch repair (MMR) genes lead to autosomal dominant inheritance of hereditary nonpolyposis colorectal cancer (HNPCC) or Lynch syndrome (Arora et al. 2017) and homologous recombination (HR) in Fanconi anaemia, Nijmegen syndrome and Bloom syndrome. These syndromes all result from chromosomal instability with the genes identified involved in aspects of DNA damage repair (Taylor 2001). Moreover, HR contributes to the repair of DSB in mammalian cells and is necessary for cell viability when exposed to ionising radiation which occurs during late radioresistant S phase of the cell cycle and the rather not radioresistant G2 phase of the cell cycle (Zhong et al. 2016). We share the opinion of other researches that partial or temporary inhibition of recombination processes merged with the radiotherapy may be an approach to cancer treatment in the nearby future.

In lung cancer malignancy, disruption of the normal cell cycle regulation is amongst the critical altered pathways due to TS exposure. TS components induce nicotine acetyl-choline receptor (nAChR) signalling, activating cell surface receptors such as β-adrenergic and EGF receptors stimulating tumour promoting cascade. This interaction facilitates tobacco-induced cancer progression, an important genomic alteration in lung cancer. It has also been evident that TS represses negative regulators of cell cycle progression including cyclin-dependent kinase (CDK) inhibitors. Two classes of CDK inhibitors are capable of arresting cells in G_1_ phase halting cell cycle progression by preventing their tumour suppressor retinoblastoma (RB) phosphorylation. Functional inactivation of RB protein induced by nAChR leads to deregulated E2F activity, corresponding with aberrant cell proliferation driving cell cycle progression and inhibiting components that arrest cell cycle (Schaal and Chellappan 2014). Inactivation of RB1 is involved in the development of NSCLC (Imai et al. 2004). Genome replication and progression through each cell cycle division is dictated by the cyclin-dependent kinase (CDK)-RB-E2F axis, the cell cycle progression transcriptional machinery. Disruptions in any of (CDK)-RB-E2F axis components lead to a heightened mitogenic E2F activity and uncontrolled proliferation (Kent and Leone 2019).

Our GSEA analysis showed significantly elevated expression of E2F genes family and its targets in LUAD tumour samples associated with TS exposure. Thus, we analysed CDK-RB-E2F genes expression, including but not limited to *AURKB*, *BUB1*, *CDCA2* and *DLGAP5*. Our findings confirmed previous reports showing that *AURKB* gene is frequently overexpressed in tumour samples of lung cancer patients, a key regulator of mitosis associated with poor prognosis (Yu et al. 2018), possibly reflecting the requirement for increased mitotic spindle genes expression for increased replication maintaining a tumorigenic phenotype (Al-Khafaji et al. 2017). Our analysis showed that *AURKB* expression is three times higher in tumour samples from smokers than never smokers. Interestingly, *AURKB* expression in newborns was found to be related to the mother’s smoking status, with observed decreased expression in offsprings of mothers who quit the use of tobacco during pregnancy (Nguyen et al. 2019). We also found the expression of *BUB1* to be 2.5 times higher in CS tumour samples. *BUB1* codes the mitotic checkpoint protein serine/threonine kinase which plays an important role in chromosome segregation (Han et al. 2015). Bidkhori et al. established that the overexpression of BUB1—involved in cell division—amongst other genes leads to tumour progression in LUAD despite of patients’ smoking status (Bidkhori et al. 2013). Both proteins AURKB and BUB1 are linked in literature with the poor prognosis of different cancers (Davidson et al. 2014; Ricke and van Deursen 2011). Another cell cycle associated gene which we found to be 2.5 times overexpressed in CS tumour samples is *CDCA2*. Shi et al. observed upregulated levels of *CDCA2* gene in vivo and in vitro in lung carcinoma cells with adjacent normal tissue. Furthermore, they noticed that knockdown of CDCA2 in LUAD tissue inhibits tumour cells by G_1_ phase arrest, therefore CDCA2 seems to play a significant role in adenocarcinoma progression (Shi et al. 2017, 2). All four (*AURKB*, *BUB1*, *DLGAP5* and *CDCA2*) described genes emerging from our analysis are regulated by the p53-DREAM pathway. DREAM is a transcriptional repressor that binds to E2F and participates in the control of all checkpoints from DNA synthesis to cytokinesis including G_1_/S, G_2_/M and spindle assembly checkpoints. According to numerous studies, downregulation of DREAM target genes promotes general loss of checkpoint control, chromosomal instability and aneuploidy of cancer cells (Engeland 2018).

Landi et al. showed that mitotic genes involved in cancer development are deregulated by TS; this applies specifically to genes that regulate the mitotic spindle formation (Landi et al. 2008). Our results run in parallel with theirs regarding *PRC1*, *MAD2L1*, *ASPM*, *RACGAP1*, *CCNA2*, *MKI67*, *KIF2C*, *TPX2*, *BIRC5*, *TTK*, *NEK2* and *CENPF*, where they were all found to be overexpressed in CS-related tumours (Supplementary Table 6). These data shed a light on characterising the molecular profile differentiating tumours in the context of TS in LUAD. It has already been shown that TS, nicotine in particular, upregulates *BIRC5* expression in NSCLC possibly inducing an early developmental stage in adenocarcinoma CS compared to NS (Hirano et al. 2015). We identified higher expression in CS of 1.5 log fold change for *BIRC5*. *TTK*, a key component of the spindle assembly checkpoint, is linked to mitosis through *EGFR* gene which is frequently altered in lung cancer (Landi et al. 2008). *ESPL1* was previously found to be overexpressed in lung adenocarcinoma smoking patients (He et al. 2018), and Zhang et al. suggested that it may be combined with other genes including *BUB1* and *E2F1* to form a regulatory network causing abnormalities of cell proliferation, transport and metabolism hence facilitating tumour progression (Zhang et al. 2016). The loss of mitotic checkpoint, chromosomal instability and aneuploidy are not only mechanisms supporting cancer development but, additionally, proliferation and metastasis. All these processes undergo the control of resistin coding by *RETN* gene. Our results show *RETN* to be upregulated in NS. Its expression also seemed to increase as the size of the tumour and clinical stage progressed and correlated with poor clinicopathological status. Zhao et al. (2018) demonstrated no correlation of resistin expression with sex, point of diagnosis, smoking or blood type in LUAD unlike in other forms of cancer (Zhao et al. 2018).

Another gene of interest to us is *CDCA5* which has also been linked to cell cycle progression and its overexpression in lung adenocarcinoma leading to tumour progression. The *CDCA5* gene encoding the sororin protein joins the cohesin complex regulating sister chromatid segregation. In mitosis, sororin undergoes phosphorylation, and protein kinases such as Cdk1/cyclinB and ERK2 regulate its dynamic localisation and function (Bidkhori et al. 2013). Thus, it interacts with the key regulatory factors ERK and cyclin E1 of G_1_/S mitotic checkpoint and in the S and G_2_/M phase ensuring accurate separation of sister chromatids by interacting with coherents and CDK1 (Wu et al. 2019). We found the expression of this gene to be 1.5 times higher in CS tumour samples compared to NS tumour samples. The E2 ubiquitin-conjugating enzyme family member UBE2C is overexpressed in 27 human cancers, possibly acting as a proto-oncogene. UBE2C is involved in mitotic cyclin B degradation, promoting cell cycle transition from M to G_1_ phase. Its overexpression leads to changes in ubiquitination; thus, it could possibly be involved in uncontrolled cell proliferation; additionally it has been identified to be associated with smoking in LUAD (Dastsooz et al. 2019), confirming our results, and drinking habits in PAAD. Oliviero et al. also identified that genes with strong and very strong correlations with *UBE2C* are involved in the cell cycle process. Of the genes they identified, we here also show that over expression of some of them are associated with smoking status in our study including *KIF18B*, *KIFC1*, *KIF4A*, *AURKB*, *TPX2*, *PLK1*, *CDK1*, *CENPA*, *CDC20*, *MYBL2*, *BUB1B*, *CCNB1*, *NCAPG*, *SKA3*, *E2F1* and *RAD51*. Of the related genes include several different roles involved in mitosis (*KIF18B*, *AURKB*, *TPX2*, *NCAPG*); regulation of cell division and DNA damage response (PLK1); cell division and driving cells into S phase (*CDK1*); cell cycle progression, survival and differentiation (*MYBL2*); mitotic checkpoint complex (*BUB1B*); and controlling the correct exit from meiosis, migration of meiotic spindle (*SKA3*). They also showed co-expression of transcription factor RAD51 involved in HR and DNA repair, and E2F1 activating genes involved in the G_1_/S phase, which was already stated, were identified to be differentially expressed in our LUAD cohort and overexpressed in our CS group. When expressed and activated in late G_*1*_ and S phase, *MYBL2* binds directly to the promoters and transactivates genes expressed in G_2_/M phase including *CCNB1*, *CDK1* and *CCNA2* (Musa et al. 2017), all genes identified in our study (Supplementary Table 6).

*SNHG6* is a novel oncogene promoting LUAD cell proliferation, migration, invasion, EMT and cell cycle progression in vitro. Findings by Liang et al. suggest that SNHG6-miR-26a-5p-E2F7 axis is critical for LUAD cell metastasis and EMT and that *E2F7* could rescue the inhibitory effects of sh-SNHG6 or miR-26a-5p (Liang et al. 2018). Our results for *E2F7* show a 1.5 log fold change, with a higher expression in CS. *E2F7* has been shown to repress the expression of genes involved in maintaining genomic stability throughout the cell cycle as well as upon induction of DNA lesions interfering with replication fork progression. It has also been shown to restrict HR through transcriptional repression of *RAD51*, also a gene identified to be overexpressed in our CS cohort. Briefly, E2F7 DNA damage response function is through transcriptional-dependent regulation of DNA repair genes (Mitxelena et al. 2018). *SCGB1A1* seems to be an important defence molecule of the lung, with anti-inflammatory function protecting the lungs from excessive inflammation. It is hypothesised that chronic cigarette smoke exposure shuts down *SCGB1A1* through an epigenetic mechanism (Zhu et al. 2015). Our results with a lower expression of 2.7-folds in CS compared to NS, run in parallel with it being one of the top hypermethylated genes in CS compared to NS in their epithelial cells (Zhu et al. 2015). We found PRG4 to be overexpressed in NS almost 6 folds with its role widely studied in modulating inflammatory responses of OA; in cancer, however, its anti-invasive effect has only been investigated in breast. In breast cancer, PRG4 inhibitor demonstrated a higher fold change difference between the early stage of breast cancer compared to controls, suppressing cell migration and invasiveness (Lee et al. 2016).

Multidrug resistance (MDR) in cancer cells could notably attenuate chemotherapy response and increase the likelihood of mortality. The overexpression of ATP-binding cassette (ABC) transporters is a major mechanism presenting MDR, resulting in increased efflux of drugs from cancer cells, thereby decreasing intracellular drug concentration (Sun et al. 2012). Additionally, it is considered that ABC transporters might also be involved in early steps of carcinogenesis (Andersen et al. 2015). Interestingly, adenoma cells present a higher level of *ABCC2* gene expression than carcinoma tissues. Furthermore, upregulation of ATP-binding cassette (ABC) transporter genes is overexpressed in bronchial tissue of chronic smokers in comparison to patients following smoking cessation (van der Deen et al. 2005). The present study identified *ABCC2* gene expression to be significantly higher, four times, in CS tumour samples than NS. It is worthy of mentioning that the expression of all genes included in ABC transporters cellular pathway were enriched in our study amongst CS tumour samples (Supplementary Table 5). Cancer treatment success is not only associated with MDR mechanisms, but specific genetic mutations may also result in increased drug sensitivity or therapeutic resistance. For instance, EGFR tyrosine kinase inhibitors (TKIs) being the first line treatment of choice for adenocarcinoma patients, T790M mutation is the major mechanism of acquired resistance (Hochmair et al. 2019). Therefore, identifying new potential cancer mutations could contribute to the development of new therapeutic strategies. Here we analysed the top mutated genes in adenocarcinoma tissue and found higher mutation count in CS population overall, with six being statistically significant (Supplementary Table 10). TP53 and EGFR mutations are common and have been identified to alter clinical outcomes, substantially if they were concurrent, activating alternative proliferative pathways that bypass EGFR as a target (Vyse and Huang 2019). The mutation of some of these genes according to literature, *EGFR*, *TP53*, *ZFHX4* and *MUC16*, is characterised and correlated with potential cancer therapeutics (Qiu et al. 2019; Aithal et al. 2018, 16; Goodwin 2018), whereas others, *RYR2*, *LRP1B* and *NAV3*, are yet to be investigated (Wang et al. 2019; Carlsson et al. 2012). We identified a key component of DNA homologous repair-*RAD51* to be overexpressed in CS. Radiation can induce *RAD51* to express and translocate between cytoplasm and nucleus (Zhong et al. 2016). A recent study in NSCLC cell lines established evidence that EGFR status demonstrates distinct radiosensitivity and DSB repair function associated with the expression and subcellular distribution of RAD51. Consequently, defective RAD51-mediated DNA repair could be a consequence of aberrant signal transduction from hypersensitive NSCLCs with EGFR mutation (Zhong et al. 2016), hence there, shedding light on possible effective role as a therapeutic in radiation resistance EGFR cells.

EMT plays an important role in tumour progression and metastatic invasion. Additionally, it has been reported to cause acquired resistance to EGFR-tyrosine kinase inhibitors, and several signalling pathways including Wnt, Notch and integrin are known to activate EMT through transcriptional repression of E-cadherins (Loh et al. 2019). A recent study by Yu et al. (2019) investigated in vitro the role of asbestos-related microenvironment on lung cancer progression, revealing an increase in proliferation and migration of lung cancer cells exposed to media from asbestos-exposed lung fibroblasts, thus highlighting the importance of the interaction of lung fibroblasts with asbestos and lung cancer metastasis (Yu et al. 2019), hence there supporting our hypothesis that the local environment is of importance for the development of specific cancer natures and imprints on the tumour’s local EMT-related features, prompting aggressiveness. Additionally Zhou et al. demonstrated that during endochondral ossification, oestrogen blocked the EMT process from the resting zone (RZ) to the proliferative zone (PZ), whereas androgen promoted MET from PZ to hypertrophic (HZ) (Zhou et al. 2015). Our results regarding EMT analysis indicate the need for further investigation of the role of androgen and oestrogen on the process. Cigarette smoking plays a vital role in promoting EMT and is associated with poor survival, cell migration and invasion in NSCLC through the deregulation of E-cadherin. Our results also show VEGF and TFG-*β* signalling pathway to be significantly enriched. TGF-β signalling pathway plays a quite important role in EMT (Xu et al. 2009). Fantozzi et al. found that VEGF-A is required for the increased tumorigenicity of cells undergoing EMT, yet not sufficient on its own. An additional angiogenic factor regulated in their expression during EMT is critical for an effective tumour initiation. Their results seem to give perception as to the mechanisms underlying increased tumourigenicity of cancer stem cells (CSC) and cells undergoing EMT in line with similar results in skin and brain tumours (Fantozzi et al. 2014). We identified collagen type X alpha 1 (COL10A1), a member of the collagen family, to be enriched in skeletal development. In gastric cancer (GC), *COL10A1* was found to possibly act as a potent co-stimulator of TGF-β1-induced EMT, where its expression was vastly elevated during GC development and progression. It also promoted cell migration and invasion via upregulation of the TGF- β1-SOX9 axis, while its knockdown slowed TGFβ1-induced EMT (Li et al. 2018). Thus similar to GC, it is worthy of elucidating the role of *COL10A1* in LUAD as it could possibly serve as a biomarker and therapeutic target.

TS is an important risk for lung and head and neck squamous carcinoma (HNSCC), where benzo[a]pyrene (B[a]P) in cigarette smoke shown evident to induce EMT-related gene expression, impacting multiple transcriptional changes in lung cancer cells. Of our statistically significant EMT genes, *TCF3* was significant for both smoking subgroups. *TCF3* is involved in the regulation of Wnt signalling pathway in embryogenesis and development (Li et al. 2017). In females, it was indeed identified to show response to smoking stimulus along with *ICOSLG*, the vascular adhesion molecule 1 *VCAM1* in B cells, emphasising the significance of B cells in the aetiology of smoking-induced diseases involving interactions between immune cells and vascular endothelium (Pan et al. 2010). However, in their study, they found its expression to be downregulated in female smokers contrary to our results where we did not separated both sexes. This is very much likely attributed to sex as well as the effect of smoking, accentuating the influence of sex on future exploratory analysis.

Yoshino et al. showed that TS promoted EMT by upregulating TWIST (Yoshino et al. 2007). Further studies by Zhu et al. investigating *TWIST* interactions with gender and smoking in patient survival revealed that both smoking and gender could modify the effects on the risk of death in HNSCC patients. Their analysis revealed that *TWIST1* links to poor survival obvious in either males or smokers than in either female or non-smokers (Zhu et al. 2017). Additionally, Stoyianni et al. used cancers of unknown primary (CUP) as a model for metastatic dissemination to study the prognostic significance of EMT. The presence of EMT phenotype significantly correlated with male gender, anaplasia and visceral metastases with strong adverse prognostic significance on patient survival. They also showed as others already did a possible role for NOTCH2 and 3 activation in regard to the induction of EMT phenotype (Stoyianni et al. 2012). Since there is almost no information regarding smoking and sex on the expression of EMT-related genes (except for *TWIST1/2*), we wanted to observe gender and smoking-induced differences correlating to the expression of EMT-related genes that could possibly allow unravelling of potential biomarkers for personalised therapy. Joint analysis of 144 EMT marker genes accounting for sex and smoking status allowed us to identify 4 statistically significant genes in all four groups (FN, MN, FC, MC) of which one was related to the NOTCH pathway (Fig. 3). Statistically significant differences between FN and FC groups included *HEY2* (*p* = 0.048), *OLFM1* (*p* < 0.05) and *STRAP* (*p* = 0.0031); for FN vs MC included *OLFM1* (*p* < 0.05) and *STRAP* (*p* = 0.0001); and, finally, for MN and MC groups was *SFRP1* (*p* = 0.0391). Overexpression of *OLFM1* was found to result in smaller metastatic and pulmonary nodules and reported as a potential target for the diagnosis and treatment of LUAD (W. Shi et al. 2016). *SFRP1* which functions as modulators of Wnt signalling is a favourable predictive and prognostic biomarker for prostate cancer (Zheng et al. 2015). While both *STRAP* and *HEY2* are involved with NOTCH signalling pathway, *STRAP* promoted the stemness of HCC through epigenetic regulation (Jin et al. 2017). In NSCLC, lncRNA *PRNCR1* upregulates *HEY2* promoting tumour progression by competitively binding miR-448 (Cheng et al. 2018).

The National Comprehensive Cancer Network (NCCN) Clinical Practice Guidelines in Oncology proposed that a patient’s sex to be a prognostic factor in LUSC, LUAD, HNSC, KIRC and KIRP. This allows to provide an overview of molecular differences between cancer patients of both sexes with distinct patterns of the effect of sex on the patient group in each cancer type. Sex-biased genes include EGFR—the key therapeutic target in LUAD—with female-biased mRNA expression possibly contributing to a higher response rate in female patients to their counterparts (Yuan et al. 2016). Our results for GSEA analysis (Supplementary file 3) for female and male LUAD patients regardless of smoking status seem to contrast those of Yang et al. in two glioblastoma (GBM) studies, where they found cell cycle signalling pathways to be critical determinants of survival in males and integrin signalling for females (Yang et al. 2019, 2017) in both molecular function and biological processes gene sets. It is interesting to find out whether these differences seem to be attributed to the effect of tobacco smoking or due to an altered mechanism in lung adenocarcinoma metabolism.

Also in relation to sex differences, we found a few of our DEGs to be overexpressed in female NS including *PLA2G2A* and *PRG4* while *REEP1* and *PLA2G2A* in male CS (Supplementary Table 8). Underexpressed genes also include *GINS4* in female NS and *WIF1* and *PRG4* in female CS. *PLA2G2A* a member of the subfamily of group II sPLA2 was overexpressed in our NS samples as part of the GnRH signalling pathway members, in both CS and NS seems to have a higher log fold change for females to males. Proteoglycan 4 (*PRG4*) appears to have an inversely proportional relationship between females and males in our CS and NS cohort. *PRG4* also seems to have a unique ability to block NFkB activation downstream from two different receptor families (CD44 and TLRs), where it might possibly be an anti-inflammatory agent (Alquraini et al. 2015). This could possibly explain its higher expression in male CS tumour samples as according to their habits they seem to use all tobacco products more often compared to females (Higgins et al. 2015). Also of interest to us were *GINS4* and *WIF1* being overexpressed in males CS compared to females. GINS4 has an essential role in DNA replication initiation and progression of DNA replication forks and the DSB repair via break induced replication (Kamada et al. 2007). While WIF1 is an inhibitor of the Wnt/β-catenin pathways, its overexpression in male CS could possibly play a role in the regulation of cell proliferation, metastasis and differentiation. That is because in osteosarcoma, WIF1 knockdown rescued proliferation, migration and invasion of osteosarcoma cells (Cai et al. 2019). *REEP1/2* has been shown to be selectively upregulated in response to DNA damage, being downstream targets of p53. Zheng el al. showed that p53 indirectly regulates endoplasmic (ER)-mitochondria contacts and Ca2+ via upregulating *REEP1/2*, *EI24* and *VDAC2*. REEP1/2 are accessory elements helping ER to expand and facilitate EI24-mediated apoptosis and overexpression of *REEP1* but not *REEP2*, promoting DNA-damage induced apoptosis (Zheng et al. 2018). This possibly explains the increased expression levels of *REEP1* in our female cohort corresponding with the hypothesised better overall survival compared to males.

Differences in the immune system and genetic architecture, whether being sex-specific or due to smoking status, will allow unravelling of epigenetic marks and shedding light on the mechanisms potentially providing scientific evidence for interventions, cancer surveillance and treatment. Pharmacokinetics and pharmacodynamics differ in individuals in terms of toxicity and efficacy; therefore, sex influences pathophysiology, clinical signs and treatment and response in cancer, making it vital to further investigate outcome implications based on potential sex differences in cancer, enhancing and personalising therapeutics. In the future, pharmacogenomic differences between sexes could have a noteworthy role in chemotherapy. An understanding of the significance of sex-specific genetics and epigenetic architecture of human disease could help reveal the existence of sex-specific protective mechanisms that could be utilised in novel treatments (Gabory et al. 2013).

Nevertheless, it should be mentioned that one of the caveats of our study was the lack of control and normal samples from LUAD patients, to help advance our understanding in determining biomarkers that are differentially expressed based on the aspects of tumour and sex of the patient due to their unavailability. Another limitation includes the inability to divide the patients into subgroups according to their age/stage due to the small number of cases meaning that prospective study would have had an insufficient number for the analysis to allow a reliable investigation. We believe that it is unfortunate that patients of certain subgroups including the elderly and those with poor performance are not considered in advanced personalised therapy as they do require specific treatment considerations in terms of targeted therapies.

## Conclusion

Here we constructed a gene co-expression network and identified 9 modules with distinct expression profiles of LUAD in CS and NS patients. One thousand one hundred fifty genes showed a visible differentiation between the subgroups. These genes were primarily involved in *cell cycle*, *replication and proliferation.* Twenty-five genes were also identified with a twofold or higher between the smoking subgroups proving some clues on the effect of smoking prevalence on LUAD. We found a contradictory pattern for EMT members between NS and CS that could be a result from direct nicotine exposure or possibly other gene interactions giving rise to a different outcome due to mutation, treatment or gender differences. Our study highlights significant differences in male and female patients, identifying new candidate genes such as *PLA2G2A* and *PRG4* that could enable a better understanding of sex-specific differences in the course of lung carcinogenesis. A thorough analysis of the biochemical processes associated with pathogenesis at the molecular level of LUAD assessing the impact of tobacco smoke or the lack of will enable a better search for “driver” genes contributing to its initiation or progression. An early on identification of prognostic molecular markers relating to tumour aggressiveness is useful since following resection, tumour metastasis is a fundamental barrier to long-term survival. Prospective studies need to include larger numbers of patients to accrue sufficient numbers of cases reliably investigating the risks associated with other exposures.

## Electronic supplementary material

ESM 1 (DOCX 4033 kb).

Supplementary File 1 (XLSX 91 kb).

Supplementary File 2 (XLSX 80 kb).

Supplementary File 3 (XLSX 105 kb).

## Data Availability

All data are already available and can be retrieved from The Cancer Genome Atlas (TCGA) database (http://gdac.broadinstitute.org/ data status of Jan 28, 2016), Genome Expression Omnibus (GEO) (https://www.ncbi.nlm.nih.gov/geo/) and cbioportal website (https://www.cbioportal.org/) for Lung Adenocarcinoma (TCGA, Firehose Legacy).

## References

[CR1] ‘CBDM Bioinformatics, Scott Davis’ (n.d.) Accessed 14 February 2020. http://cbdm.hms.harvard.edu/LabMembersPges/SD.html

[CR2] ‘GraphPad Prism7 User Guide’ (n.d.) Accessed 14 February 2020. https://www.graphpad.com/guides/prism/7/user-guide/index.htm?citing_graphpad_prism.htm

[CR3] Aithal A, Rauth S, Kshirsagar P, Shah A, Lakshmanan I, Junker WM, Jain M, Ponnusamy MP, Batra SK (2018). MUC16 as a novel target for cancer therapy. Expert Opin Ther Targets.

[CR4] Al-Khafaji ASK, Marcus MW, Davies MPA, Risk JM, Shaw RJ, Field JK, Liloglou T (2017). AURKA MRNA expression is an independent predictor of poor prognosis in patients with non-small cell lung cancer. Oncol Lett.

[CR5] Alquraini A, Garguilo S, D’Souza G, Zhang LX, Schmidt TA, Jay GD, Elsaid KA (2015) The interaction of lubricin/proteoglycan 4 (PRG4) with toll-like receptors 2 and 4: an anti-inflammatory role of PRG4 in synovial fluid. Arthritis Res Ther 17. 10.1186/s13075-015-0877-x 10.1186/s13075-015-0877-x PMC4672561 26643105

[CR6] Andersen V, Vogel LK, Kopp TI, Sæbø M, Nonboe AW, Hamfjord J, Kure EH, Vogel U (2015) High ABCC2 and low ABCG2 gene expression are early events in the colorectal adenoma-carcinoma sequence. PLoS One 10(3). 10.1371/journal.pone.0119255 10.1371/journal.pone.0119255 PMC4368545 25793771

[CR7] Arora S, Huwe PJ, Sikder R, Shah M, Browne AJ, Lesh R, Nicolas E (2017). Functional analysis of rare variants in mismatch repair proteins augments results from computation-based predictive methods. Cancer Biol Ther.

[CR8] Babicki S, Arndt D, Marcu A, Liang Y, Grant JR, Maciejewski A, Wishart DS (2016). Heatmapper: web-enabled heat mapping for all. Nucleic Acids Res.

[CR9] Bhopal A, Peake MD, Gilligan D, Cosford P (2019). Lung cancer in never-smokers: a hidden disease. J R Soc Med.

[CR10] Bidkhori G, Narimani Z, Ashtiani SH, Moeini A, Nowzari-Dalini A, Masoudi-Nejad A (2013) Reconstruction of an integrated genome-scale co-expression network reveals key modules involved in lung adenocarcinoma. PLoS One 8(7). 10.1371/journal.pone.0067552 10.1371/journal.pone.0067552 PMC3708931 23874428

[CR11] Brionne A, Juanchich A, Hennequet-Antier C (2019). ViSEAGO: a bioconductor package for clustering biological functions using gene ontology and semantic similarity. BioData Min.

[CR12] Cai W, Xu Y, Yin J, Zuo W, Zhen S (2019). MiR-552-5p facilitates osteosarcoma cell proliferation and metastasis by targeting WIF1. Exp Ther Med.

[CR13] Carlsson E, Ranki A, Sipilä L, Karenko L, Abdel-Rahman WM, Ovaska K, Siggberg L (2012). Potential role of a navigator gene NAV3 in colorectal cancer. Br J Cancer.

[CR14] Chapman AM, Sun KY, Ruestow P, Cowan DM, Madl AK (2016). Lung cancer mutation profile of EGFR, ALK, and KRAS: meta-analysis and comparison of never and ever smokers. Lung Cancer.

[CR15] Cheng D, Bao C, Zhang X, Lin X, Huang H, Liang Z (2018). LncRNA PRNCR1 interacts with HEY2 to abolish MiR-448-mediated growth inhibition in non-small cell lung cancer. Biomed Pharmacother.

[CR16] Couraud S, Zalcman G, Milleron B, Morin F, Souquet P-J (2012). Lung cancer in never smokers – a review. Eur J Cancer.

[CR17] Couraud S, Souquet P-J, Paris C, Dô P, Doubre H, Pichon E, Dixmier A (2015). BioCAST/IFCT-1002: epidemiological and molecular features of lung Cancer in never-smokers. Eur Respir J.

[CR18] Dastsooz H, Cereda M, Donna D, Oliviero S (2019). A comprehensive bioinformatics analysis of UBE2C in cancers. Int J Mol Sci.

[CR19] Davidson B, Nymoen DA, Elgaaen BV, Tropé CG, Kærn J, Reich R, Hetland TE, Falkenthal, Anne Cathrine Staff (2014). BUB1 MRNA is significantly co-expressed with AURKA and AURKB MRNA in advanced-stage ovarian serous carcinoma. Virchows Arch.

[CR20] Dias M, Linhas R, Campainha S, Conde S, Barroso A (2017). Lung cancer in never-smokers – what are the differences?. Acta Oncol.

[CR21] Doll R, Peto R (1978). Cigarette smoking and bronchial carcinoma: dose and time relationships among regular smokers and lifelong non-smokers. J Epidemiol Community Health.

[CR22] Dorak MT, Karpuzoglu E (2012) Gender Differences in Cancer Susceptibility: An Inadequately Addressed Issue. Front Genet 3. 10.3389/fgene.2012.00268 10.3389/fgene.2012.00268 PMC3508426 23226157

[CR23] Engeland K (2018). Cell cycle arrest through indirect transcriptional repression by P53: I have a DREAM. Cell Death Differ.

[CR24] Fantozzi A, Gruber DC, Pisarsky L, Heck C, Kunita A, Yilmaz M, Meyer-Schaller N (2014). VEGF-mediated angiogenesis links EMT-induced cancer stemness to tumor initiation. Cancer Res.

[CR25] Ferlay J, Shin H-R, Bray F, Forman D, Mathers C, Parkin DM (2010). Estimates of worldwide burden of cancer in 2008: GLOBOCAN 2008. Int J Cancer.

[CR26] Field RW, Withers BL (2012) Occupational and environmental causes of lung cancer. Clin Chest Med 33(4). 10.1016/j.ccm.2012.07.001 10.1016/j.ccm.2012.07.001 PMC3875302 23153609

[CR27] Flanders WD, Lally CA, Zhu B-P, Jane Henley S, Thun MJ (2003). Lung cancer mortality in relation to age, duration of smoking, and daily cigarette consumption: results from cancer prevention study II. Cancer Res.

[CR28] Gabory A, Roseboom TJ, Moore T, Moore LG, Junien C (2013). Placental contribution to the origins of sexual dimorphism in health and diseases: sex chromosomes and epigenetics. Biol Sex Differ.

[CR29] Goodwin J (2018) ‘Siteman investment program awards $2.1 million in cancer research grants’. *Siteman Cancer Center* (blog). 15 June 2018. https://siteman.wustl.edu/siteman-investment-program-awards-2-1-million-in-cancer-research-grants/

[CR30] Gridelli C, Rossi A, Carbone DP, Guarize J, Karachaliou N, Mok T, Petrella F, Spaggiari L, Rosell R (2015). Non-small-cell lung cancer. Nat Rev Dis Primers.

[CR31] Han JY, Han YK, Park G-Y, Kim SD, Lee CG (2015). Bub1 is required for maintaining Cancer stem cells in breast Cancer cell lines. Sci Rep.

[CR32] He X, Cheng Z, Shi C, Quqin L (2018). Meta-analysis of MRNA expression profiles to identify differentially expressed genes in lung adenocarcinoma tissue from smokers and non-smokers. Oncol Rep.

[CR33] Higgins ST, Kurti AN, Redner R, White TJ, Gaalema DE, Roberts ME, Doogan NJ (2015). A literature review on prevalence of gender differences and intersections with other vulnerabilities to tobacco use in the United States, 2004–2014. Prev Med.

[CR34] Hirano H, Maeda H, Yamaguchi T, Yokota S, Mori M, Sakoda S (2015). Survivin expression in lung cancer: association with smoking, histological types and pathological stages. Oncol Lett.

[CR35] Hirsch FR, Scagliotti GV, Mulshine JL, Kwon R, Curran WJ, Wu Y-L, Paz-Ares L (2017). Lung Cancer: current therapies and new targeted treatments. Lancet.

[CR36] Hochmair MJ, Buder A, Schwab S, Burghuber OC, Prosch H, Hilbe W, Cseh A, Fritz R, Filipits M (2019). Liquid-biopsy-based identification of EGFR T790M mutation-mediated resistance to afatinib treatment in patients with advanced EGFR mutation-positive NSCLC, and subsequent response to osimertinib. Target Oncol.

[CR37] Imai MA, Oda Y, Oda M, Nakanishi I, Kawahara E (2004). Overexpression of E2F1 associated with LOH at RB locus and hyperphosphorylation of RB in non-small cell lung carcinoma. J Cancer Res Clin Oncol.

[CR38] Jin L, Trung V, Yuan G, Datta PK (2017). STRAP promotes stemness of human colorectal cancer via epigenetic regulation of the NOTCH pathway. Cancer Res.

[CR39] Jolliffe IT, Cadima J (2016). Principal component analysis: a review and recent developments. Philos Trans R Soc A Math Phys Eng Sci.

[CR40] Kamada K, Kubota Y, Arata T, Shindo Y, Hanaoka F (2007). Structure of the human GINS complex and its assembly and functional interface in replication initiation. Nat Struct Mol Biol.

[CR41] Kenfield SA (2008). Smoking and smoking cessation in relation to mortality in women. JAMA.

[CR42] Kent LN, Leone G (2019). The broken cycle: E2F dysfunction in Cancer. Nat Rev Cancer.

[CR43] Landi MT, Dracheva T, Rotunno M, Figueroa JD, Liu H, Dasgupta A, Mann FE (2008). ‘Gene Expression Signature of Cigarette Smoking and Its Role in Lung Adenocarcinoma Development and Survival’. Edited by Dawn Albertson. PLoS One.

[CR44] Langfelder P, Horvath S (2008). WGCNA: an R package for weighted correlation network analysis. BMC Bioinformatics.

[CR45] Lê S, Josse J, Husson F (2008) FactoMineR : an *R* package for multivariate analysis. J Stat Softw 25(1). 10.18637/jss.v025.i01

[CR46] Lee YJ, Kim J-H, Kim SK, Ha S-J, Mok TS, Mitsudomi T, Cho BC (2011). Lung cancer in never smokers: change of a mindset in the molecular era. Lung Cancer.

[CR47] Lee C-S, Taib NAM, Ashrafzadeh A, Fadzli F, Harun F, Rahmat K, Hoong SM, Abdul-Rahman PS, Hashim OH (2016). ‘Unmasking Heavily O-Glycosylated Serum Proteins Using Perchloric Acid: Identification of Serum Proteoglycan 4 and Protease C1 Inhibitor as Molecular Indicators for Screening of Breast Cancer’. Edited by Roger Chammas. PLoS One.

[CR48] Li H, Yang S, Zhang Y, Gao N, Deng X, Sheng X (2017). MicroRNA-138 is a potential biomarker and tumor suppressor in human cervical carcinoma by reversely correlated with TCF3 gene. Gynecol Oncol.

[CR49] Li T, Huang H, Shi G, Zhao L, Li T, Zhang Z, Liu R (2018). TGF-Β1-SOX9 axis-inducible COL10A1 promotes invasion and metastasis in gastric cancer via epithelial-to-mesenchymal transition. Cell Death Dis.

[CR50] Liang R, Xiao G, Wang M, Li X, Li Y, Hui ZQ, Sun X (2018). SNHG6 functions as a competing endogenous RNA to regulate E2F7 expression by sponging MiR-26a-5p in lung adenocarcinoma. Biomed Pharmacother.

[CR51] Loh C-Y, Chai JY, Tang TF, Wong WF, Sethi G, Shanmugam MK, Chong PP, Looi CY (2019) The E-cadherin and N-cadherin switch in epithelial-to-mesenchymal transition: signaling, therapeutic implications, and challenges. Cells 8(10). 10.3390/cells8101118 10.3390/cells8101118 PMC6830116 31547193

[CR52] Mahmood MQ, Ward C, Muller HK, Sohal SS, Walters EH (2017). Epithelial Mesenchymal Transition (EMT) and Non-Small Cell Lung Cancer (NSCLC): A Mutual Association with Airway Disease. Med Oncol.

[CR53] Midha A, Dearden S, McCormack R (2015). EGFR mutation incidence in non-small-cell lung cancer of adenocarcinoma histology: a systematic review and global map by ethnicity (MutMapII). Am J Cancer Res.

[CR54] Mitxelena J, Apraiz A, Vallejo-Rodríguez J, García-Santisteban I, Fullaondo A, Alvarez-Fernández M, Malumbres M, Zubiaga AM (2018). An E2F7-dependent transcriptional program modulates DNA damage repair and genomic stability. Nucleic Acids Res.

[CR55] Musa J, Aynaud M-M, Mirabeau O, Delattre O, Grünewald TGP (2017). MYBL2 (B-Myb): a central regulator of cell proliferation, cell survival and differentiation involved in tumorigenesis. Cell Death Dis.

[CR56] Nagathihalli NS, Massion PP, Gonzalez AL, Lu P, Datta PK (2012). Smoking induces epithelial-to-mesenchymal transition in non-small cell lung cancer through HDAC-mediated downregulation of E-cadherin. Mol Cancer Ther.

[CR57] Nguyen T, Li GE, Chen H, Cranfield CG, McGrath KC, Gorrie CA (2019). Neurological effects in the offspring after switching from tobacco cigarettes to E-cigarettes during pregnancy in a mouse model. Toxicol Sci.

[CR58] Paik PK, Arcila ME, Fara M, Sima CS, Miller VA, Kris MG, Ladanyi M, Riely GJ (2011). Clinical characteristics of patients with lung adenocarcinomas harboring BRAF mutations. J Clin Oncol Off J Am Soc Clin Oncol.

[CR59] Pan F, Yang T-L, Chen X-D, Chen Y, Gao G, Liu Y-Z, Pei Y-F (2010). Impact of female cigarette smoking on circulating B cells in vivo: the suppressed ICOSLG, TCF3, and VCAM1 gene functional network may inhibit normal cell function. Immunogenetics.

[CR60] Pezzuto A, Citarella F, Croghan I, Tonini G (2019). The effects of cigarette smoking extracts on cell cycle and tumor spread: novel evidence. Future Sci OA.

[CR61] Pfeifer GP, Denissenko MF, Olivier M, Tretyakova N, Hecht SS, Hainaut P (2002). Tobacco smoke carcinogens, DNA damage and P53 mutations in smoking-associated cancers. Oncogene.

[CR62] Pirie K, Peto R, Green J, Reeves GK, Beral V, for the Million Women Study Collaborators (2016). Lung cancer in never smokers in the UK million women study: lung cancer in never smokers. Int J Cancer.

[CR63] Qiu Z, Lin A, Li K, Lin W, Wang Q, Wei T, Zhu W, Luo P, Zhang J (2019). A novel mutation panel for predicting etoposide resistance in small-cell lung cancer. Drug Des Devel Ther.

[CR64] Qu Y-L, Liu J, Zhang L-X, Wu C-M, Chu A-J, Wen B-L, Ma C et al (2017) Asthma and the Risk of lung Cancer: a meta-analysis. Oncotarget 8(7). 10.18632/oncotarget.14595 10.18632/oncotarget.14595 PMC5355290 28086224

[CR65] Ricke RM, van Deursen JM (2011). Aurora B Hyperactivation by Bub1 Overexpression Promotes Chromosome Missegregation. Cell Cycle.

[CR66] Schaal C, Chellappan SP (2014). Nicotine-mediated cell proliferation and tumor progression in smoking-related cancers. Mol Cancer Res.

[CR67] Shannon P (2003). Cytoscape: a software environment for integrated models of biomolecular interaction networks. Genome Res.

[CR68] Shi W, Ye Z, Zhuang L, Li Y, Shuai W, Zuo Z, Mao X (2016). Olfactomedin 1 negatively regulates NF-ΚB signalling and suppresses the growth and metastasis of colorectal cancer cells: OLFM1 inhibits NF-ΚB and CRC. J Pathol.

[CR69] Shi R, Zhang C, Wu Y, Wang X, Sun Q, Sun J, Xia W et al (2017) CDCA2 promotes lung adenocarcinoma cell proliferation and predicts poor survival in lung adenocarcinoma patients. Oncotarget 8(12). 10.18632/oncotarget.15519 10.18632/oncotarget.15519 PMC5386720 28423619

[CR70] Stoyianni A, Goussia A, Pentheroudakis G, Siozopoulou V, Ioachim E, Krikelis D, Golfinopoulos V et al (2012) Immunohistochemical study of the epithelial-mesenchymal transition phenotype in cancer of unknown primary: incidence, Correlations and Prognostic Utility. Anticancer Res 9 22493359

[CR71] Subramanian A, Tamayo P, Mootha VK, Mukherjee S, Ebert BL, Gillette MA, Paulovich A (2005). Gene set enrichment analysis: a knowledge-based approach for interpreting genome-wide expression profiles. Proc Natl Acad Sci.

[CR72] Sun Y-L, Patel A, Kumar P, Chen Z-S (2012). Role of ABC transporters in cancer chemotherapy. Chin J Cancer.

[CR73] Takamochi K, Oh S, Suzuki K (2013). Differences in EGFR and KRAS mutation spectra in lung adenocarcinoma of never and heavy smokers. Oncol Lett.

[CR74] Taylor AMR (2001). Chromosome instability syndromes. Best Pract Res Clin Haematol.

[CR75] van der Deen M, de Vries EGE, Timens W, Scheper RJ, Timmer-Bosscha H, Postma DS (2005). ATP-binding cassette (ABC) transporters in normal and pathological lung. Respir Res.

[CR76] Vogelstein B, Papadopoulos N, Velculescu VE, Zhou S, Diaz LA, Kinzler KW (2013). Cancer genome landscapes. Science.

[CR77] Vyse S, Huang PH (2019) Targeting EGFR exon 20 insertion mutations in non-small cell lung cancer. Signal Transduct Target Ther 4(March). 10.1038/s41392-019-0038-9 10.1038/s41392-019-0038-9 PMC6405763 30854234

[CR78] Wang C, Liang H, Lin C, Li F, Xie G, Qiao S, Shi X (2019). Molecular subtyping and prognostic assessment based on tumor mutation burden in patients with lung adenocarcinomas. Int J Mol Sci.

[CR79] Winterhalter C, Widera P, Krasnogor N (2014). JEPETTO: a Cytoscape plugin for gene set enrichment and topological analysis based on interaction networks. Bioinformatics.

[CR80] Wu Q, Zhang B, Sun Y, Xu R, Hu X, Ren S, Ma Q (2019). Identification of novel biomarkers and candidate small molecule drugs in non-small-cell lung cancer by integrated microarray analysis. Onco Targets Ther.

[CR81] Xu J, Lamouille S, Derynck R (2009). TGF-β-induced epithelial to mesenchymal transition. Cell Res.

[CR82] Yang W, Warrington NM, Taylor SJ, Carrasco E, Singleton KW, Wu N, Lathia JD et al (2017) Clinically Important Sex Differences in GBM Biology Revealed by Analysis of Male and Female Imaging, Transcriptome and Survival Data. BioRxiv 232744. 10.1101/232744

[CR83] Yang W, Warrington NM, Taylor SJ, Whitmire P, Carrasco E, Singleton KW, Wu N (2019). Sex Differences in GBM Revealed by Analysis of Patient Imaging, Transcriptome, and Survival Data. Sci Transl Med.

[CR84] Yano T, Miura N, Takenaka T, Haro A, Okazaki H, Ohba T, Kouso H, Kometani T, Shoji F, Maehara Y (2008). Never-smoking nonsmall cell lung cancer as a separate entity: clinicopathologic features and survival. Cancer.

[CR85] Yoshida K, Gowers KHC, Lee-Six H, Chandrasekharan DP, Coorens T, Maughan EF, Beal K et al (2020) Tobacco smoking and somatic mutations in human bronchial epithelium. Nature. 10.1038/s41586-020-1961-1 10.1038/s41586-020-1961-1 PMC7021511 31996850

[CR86] Yoshino I, Kometani T, Shoji F, Osoegawa A, Ohba T, Kouso H, Takenaka T, Yohena T, Maehara Y (2007). Induction of epithelial-mesenchymal transition-related genes by benzo[a]pyrene in lung cancer cells. Cancer.

[CR87] Yu G, Li F, Qin Y, Bo X, Wu Y, Wang S (2010). GOSemSim: an R package for measuring semantic similarity among GO terms and gene products. Bioinformatics.

[CR88] Yu JJ, Zhou J, Xu F, Bai W, Zhang W (2018). High expression of Aurora-B is correlated with poor prognosis and drug resistance in non-small cell lung cancer. Int J Biol Markers.

[CR89] Yu S, Choi H-H, Kim IW, Kim T-J (2019). Conditioned medium from asbestos-exposed fibroblasts affects proliferation and invasion of lung cancer cell lines. PLoS One.

[CR90] Yuan Y, Liu L, Hu C, Wang Y, Xu Y, Mao H, Li J (2016). Comprehensive characterization of molecular differences in cancer between male and female patients. Cancer Cell.

[CR91] Zhang C, Min L, Zhang L, Ma Y, Yang Y, Shou C (2016). Combined analysis identifies Six genes correlated with augmented malignancy from non-small cell to small cell lung Cancer. Tumor Biol.

[CR92] Zhao CC, Chen J, Niu RF, Liu Y, Zhang CG (2018) Increased Resistin suggests poor prognosis and promotes development of lung adenocarcinoma. Oncol Rep. 10.3892/or.2018.6736 10.3892/or.2018.6736 PMC6196645 30272365

[CR93] Zheng L, Sun D, Fan W, Zhang Z, Li Q, Jiang T (2015). ‘Diagnostic value of SFRP1 as a favorable predictive and prognostic biomarker in patients with prostate cancer’. Edited by Craig N Robson. PLoS One.

[CR94] Zheng P, Chen Q, Tian X, Qian N, Chai P, Liu B, Hu J (2018). DNA damage triggers tubular endoplasmic reticulum extension to promote apoptosis by facilitating ER-mitochondria signaling. Cell Res.

[CR95] Zhong X, Luo G, Zhou X, Luo W, Wu X, Zhong R, Wang Y, Xu F, Wang J (2016). Rad51 in regulating the Radiosensitivity of non-small cell lung cancer with different epidermal growth factor receptor mutation status: Rad51 and Radiosensitivity in NSCLC. Thorac Cancer.

[CR96] Zhou S, Shen Y, Wang L, Li P (2015). Epithelial-mesenchymal transition and mesenchymal-epithelial transition response during differentiation of growth-plate chondrocytes in endochondral ossification. Int J Clin Exp Med.

[CR97] Zhu L, Di PYP, Wu R, Pinkerton KE, Chen Y (2015). ‘Repression of CC16 by Cigarette Smoke (CS) Exposure’. Edited by Hong Wei Chu. PLoS One.

[CR98] Zhu Y, Zhang W, Wang P (2017). Smoking and gender modify the effect of TWIST on patient survival in head and neck squamous carcinoma. Oncotarget.

