# Supplementary material for: Differential expression of lung adenocarcinoma transcriptome with signature of tobacco exposure
Source: J Appl Genet. 2020 Jun 20;61(3):421–37. doi: 10.1007/s13353-020-00569-1 (PMC7413900; doi:10.1007/s13353-020-00569-1)
Supplement: Supplementary file 1 — (DOCX 4033 kb). [file 13353_2020_569_MOESM1_ESM.docx]

**Supplementary Data for Manuscript Title:**”Differential expression of Lung adenocarcinoma transcriptome with signature of tobacco exposure”

Raneem Y. Hammouz^1^†, Joanna K. Kostanek^1^†, Aleksandra Dudzisz^1^†, Piotr Witas^1^, Magdalena Orzechowska^1^ and Andrzej K. Bednarek^1^*

^1^ Department of Molecular Carcinogenesis, Medical University of Lodz, Lodz, 90-752, Poland

† Co-first authors

* Correspondence: Andrzej K. Bednarek (ORCID:0000-0002-4570-4154) , Zeligowskiego 7/9, 90-752 Lodz, tel: +48 42 639 31 80;
fax: +48 42 639 31 80; email: [andrzej.bednarek@umed.lodz.pl](mailto:andrzej.bednarek@umed.lodz.pl)

| **Non-smokers** | | | |
| --- | --- | --- | --- |
| **Parameter** | **Total** | **Males** | **Females** |
| **Quantity** | 54 | 13 | 41 |
| **Median age (range)** |  | 60.5 | 68 |
| **Stage** |  |  |  |
| **I** |  | 6 | 23 |
| **II** |  | 5 | 12 |
| **III** |  | - | 5 |
| **IV** |  | 2 | 1 |
| **NA** |  | - | - |
| **alive** |  | **13** | **35** |
| **dead** |  |  | **6** |
|  |  |  |  |
| **Current Smokers** | | | |
| **Quantity** | 92 | 59 | 33 |
| **Median age (range)** |  | 61 | 61 |
| **Stage** |  |  |  |
| **I** |  | 30 | 15 |
| **II** |  | 16 | 11 |
| **III** |  | 8 | 6 |
| **IV** |  | 4 | 1 |
| **NA** |  | 1 | - |
| **alive** |  | **46** | **24** |
| **dead** |  | **13** | **9** |

**Supplementary Table 1.** LUAD patient cohort clinical information.

**(21)**

**Supplementary Figure 1**. Unsupervised hierarchical clustering of LUAD patient tumour samples (**A)** for non-smokers, (**B)** for current smokers, dividing each into 3 groups.


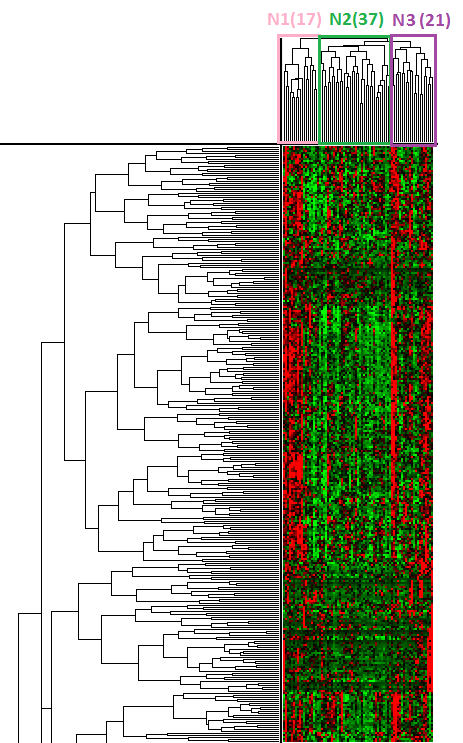

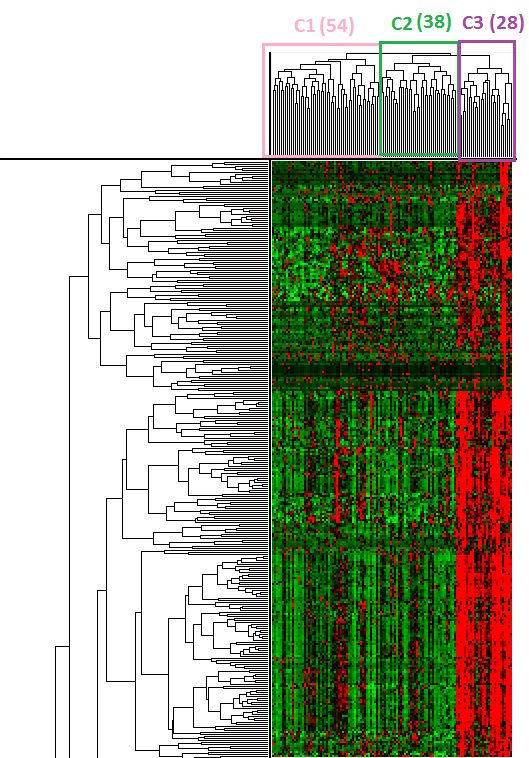


**A**

**B**


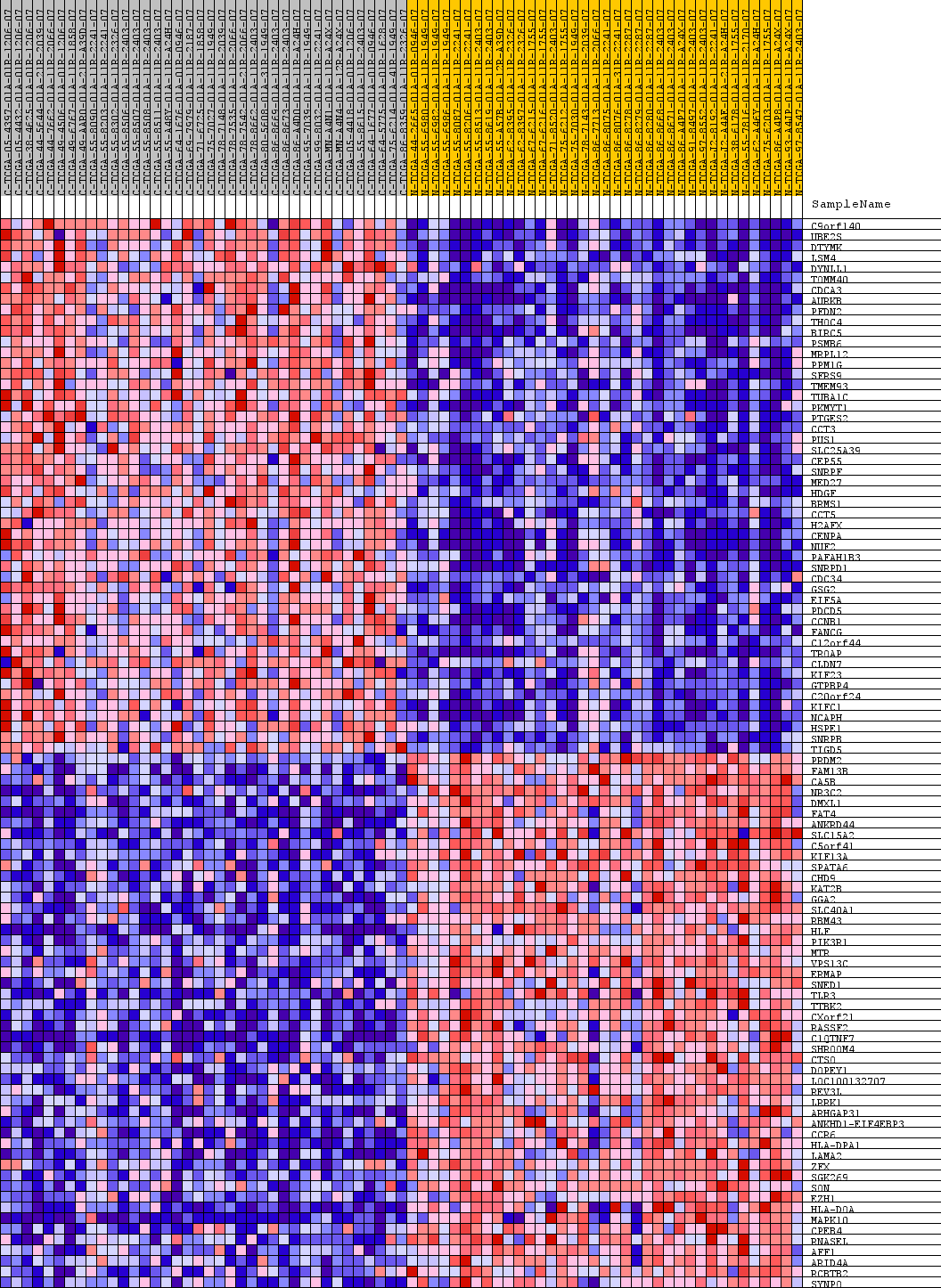


**Supplementary Figure 2**. Heatmap of the most differentially expressed genes in current smokers versus non-smokers identified by phenotypic comparision using weighted tTest metrics. Subgroup C2 versus subgroup N2 LUAD patient gene expression profiles are molecularly distinct using Gene Set Enrichment Analysis (GSEA).


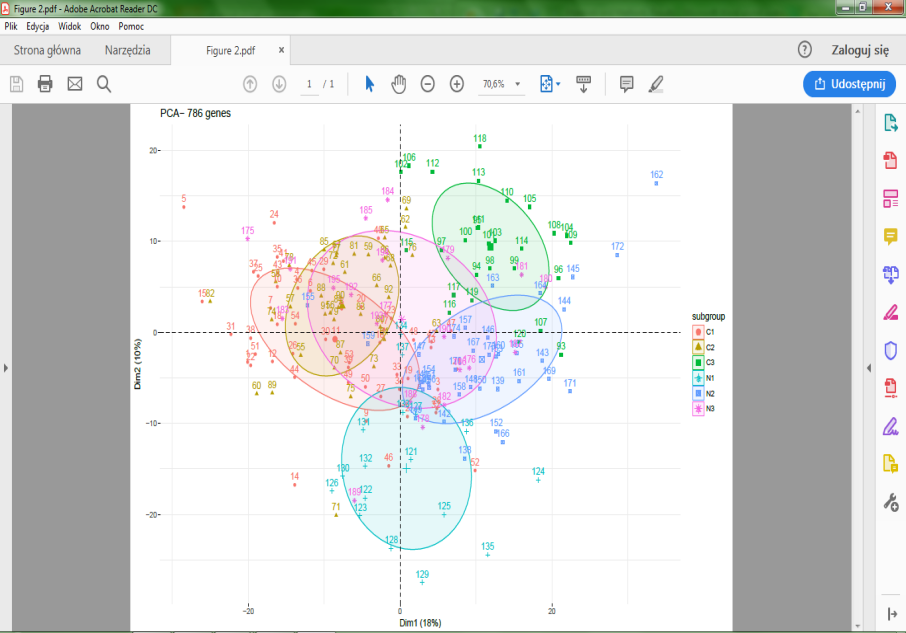


**Supplementary Figure 3.** Principle component analysis performed on expression data for 786 genes for all 6 LUAD subgroups. Subgroups clusters are represented on the factorial plan by coloured ellipses reflecting their association.

**A**


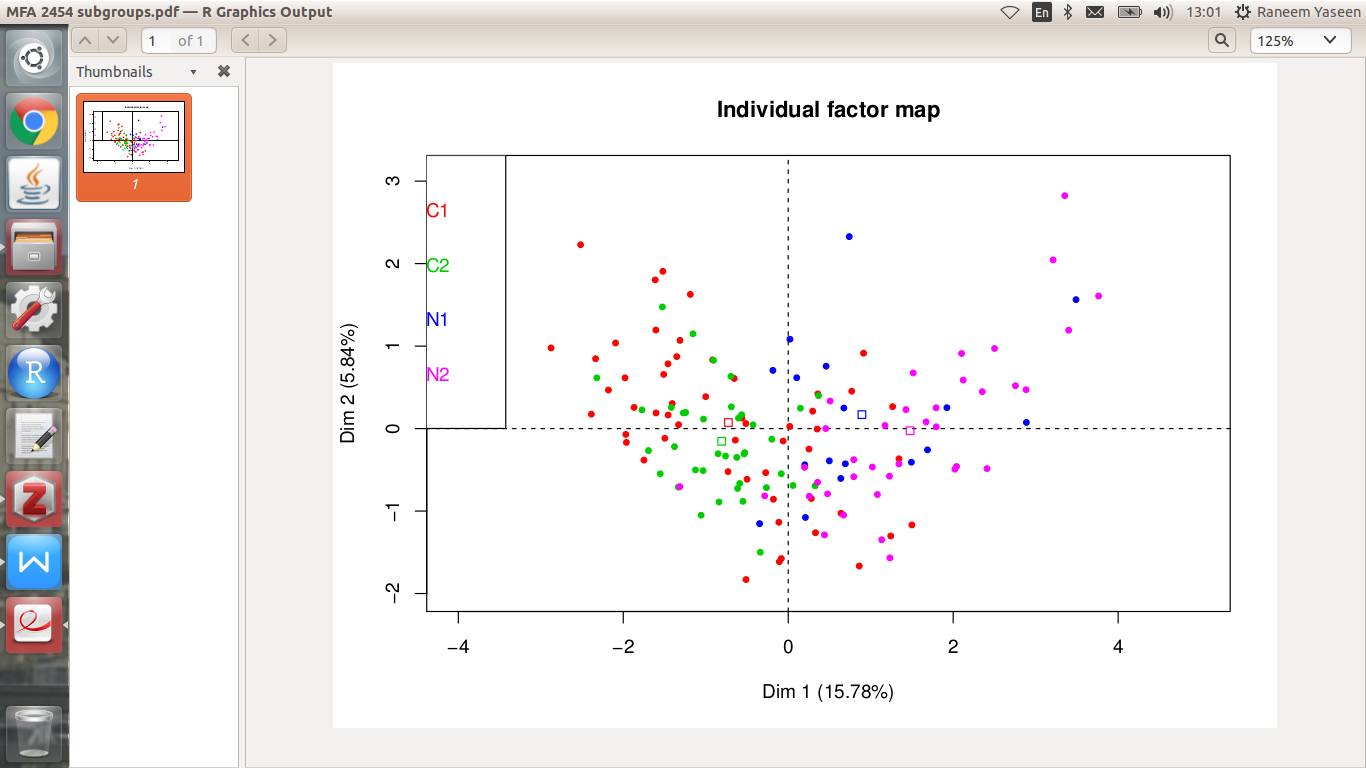

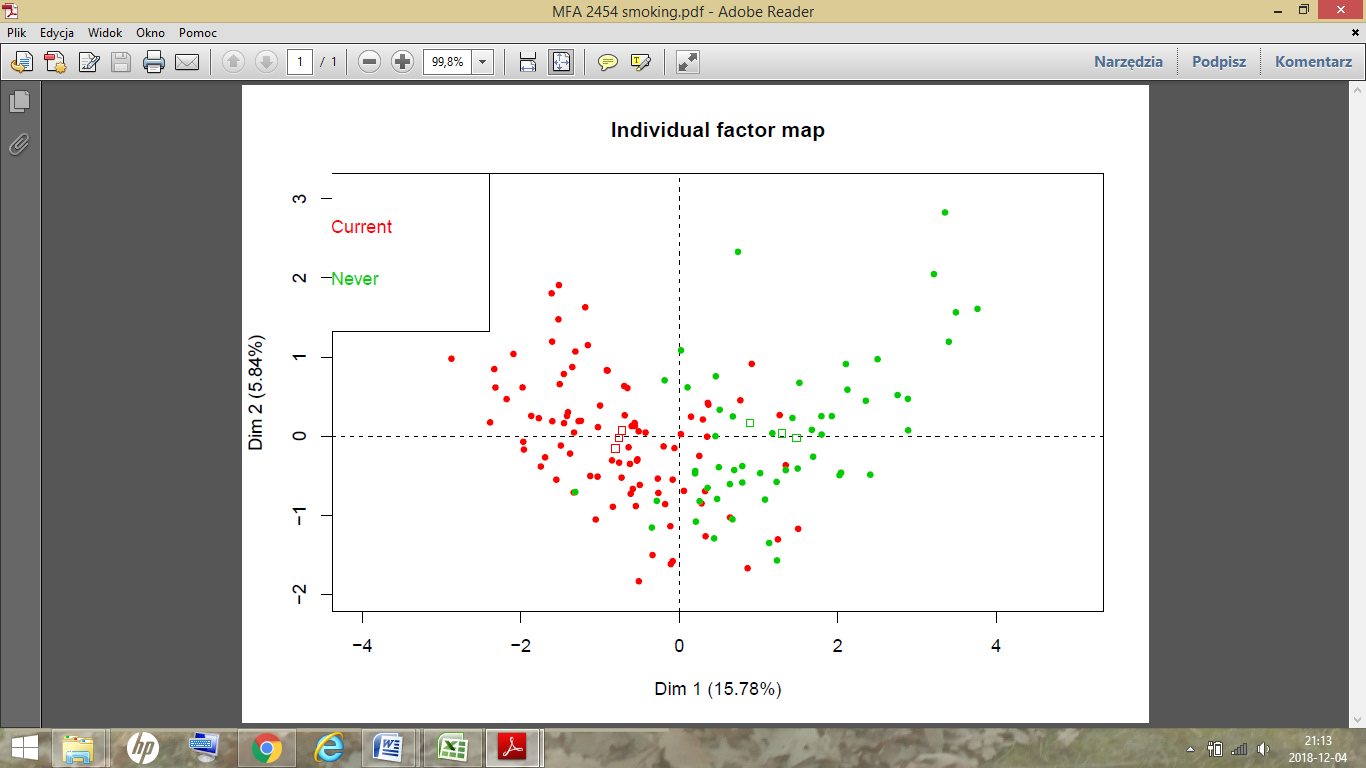


**B**

**Supplementary Figure 4.** Multiple factor analysis showing different pattern for current and non-smokers **(A)** all four groups individually **(B)** CS vs NS combined.


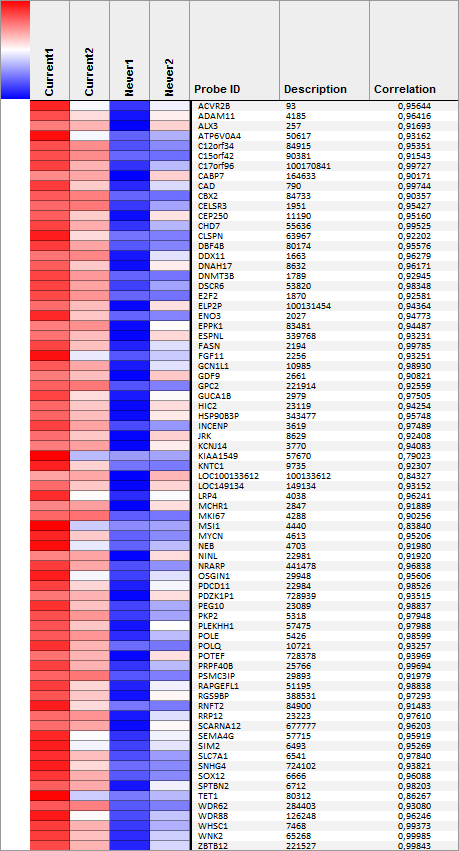


**Supplementary Figure 5.** One of the cluster showing differentiation between current and non-smokers tumour samples.

**
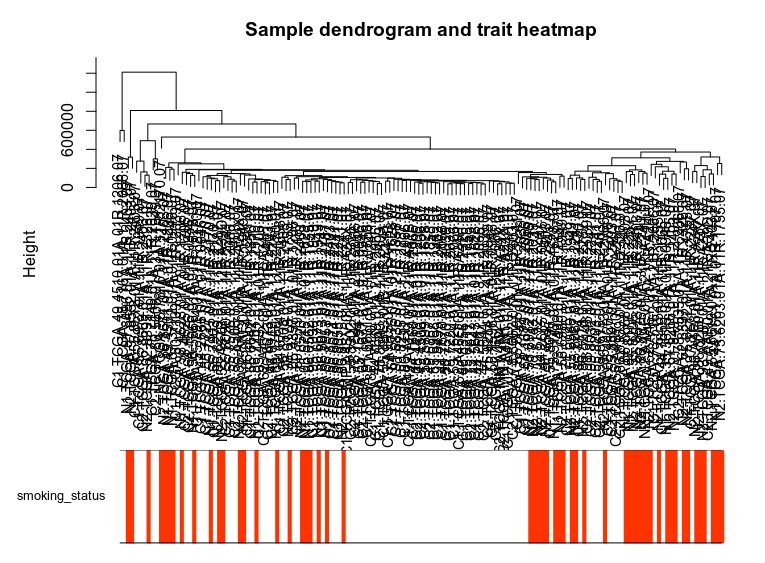
**

**Supplementary Figure 6.** Sample clustering to detect outliers. All the samples were in the clusters, all samples have passed the cuts.


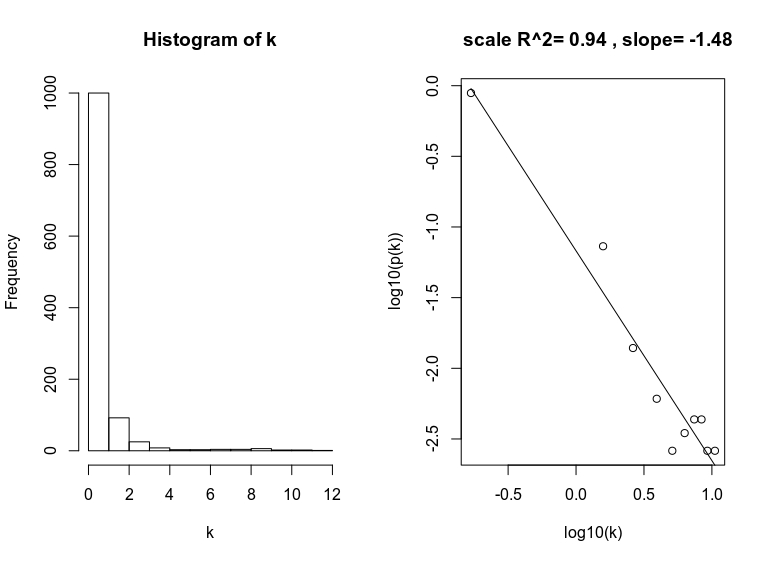

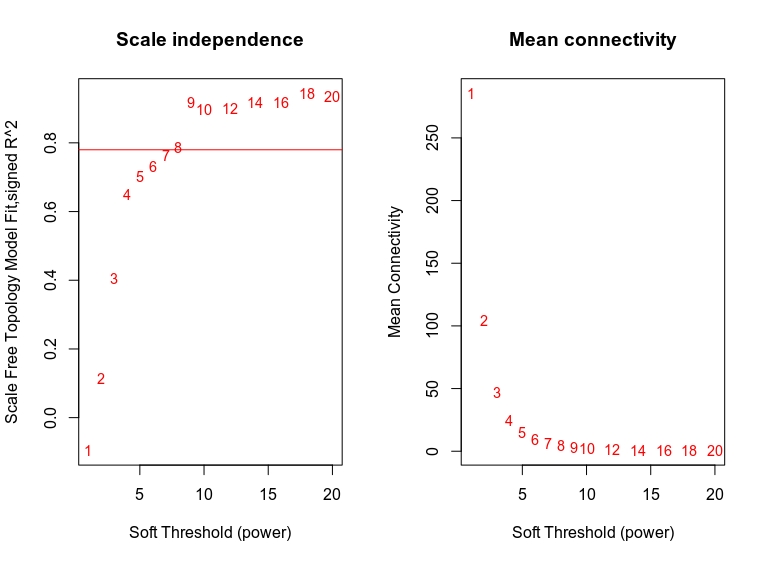


**B**

**A**

**D**

**C**

**Supplementary Figure 7.** Determination of soft-thresholding power in the weighted gene co-expression network analysis (WG). **(A)** Analysis of scale-free fit index for various soft-thresholding powers (β). **(B)** Analysis of mean connectivity for various soft-thresholding powers. **(C)** Linear model fitting of R 2 index showed good quality of fit. **(D)** Frequency distribution of connectivity.

**A**


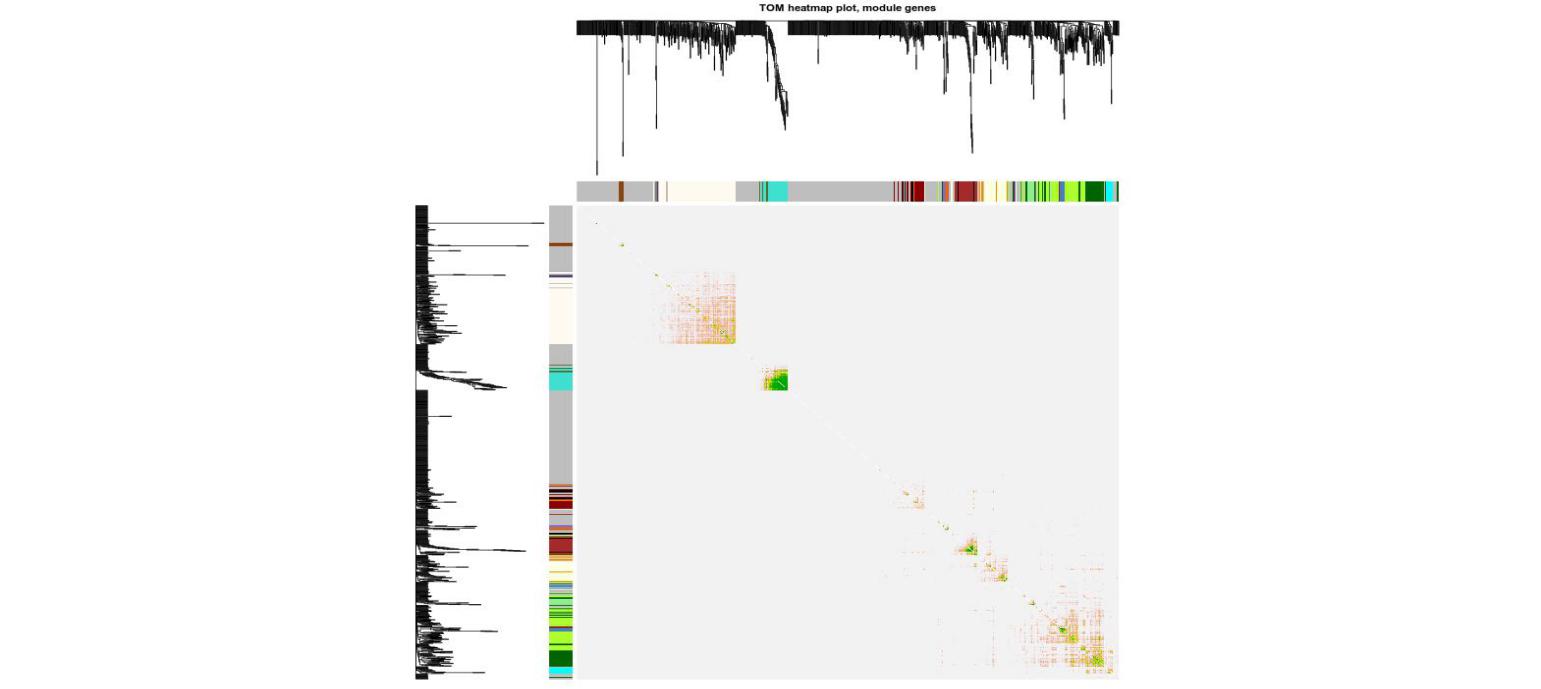

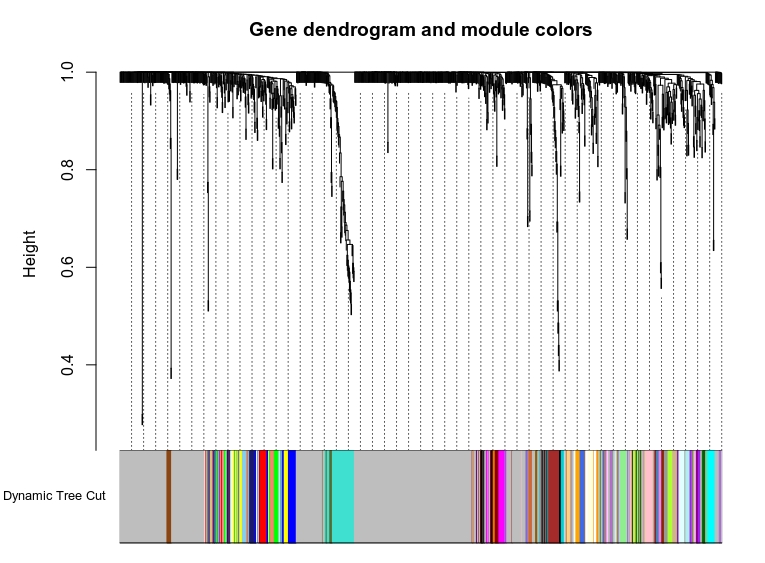


**B**

**Supplementary Figure 8. A)** Cluster dendrogram depicting WGCNA gene modules with dissimilarity based on topological overlap with assigned module colors. Following this, 22 co-expression modules were constructed shown in different color. **B)** Visualizing the gene network using a heatmap plot. The heatmap depicts the Topological Overlap Matrix (TOM) among all genes in the analysis.

**
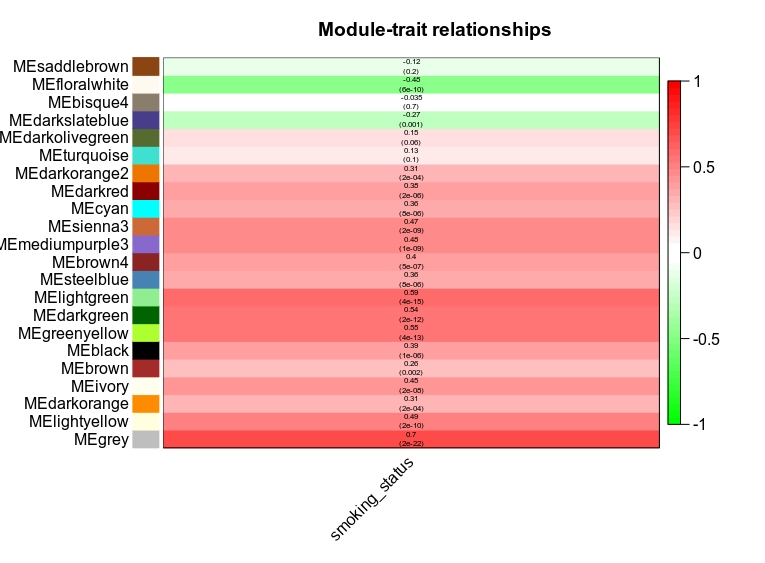
**

**A**

**B**

**
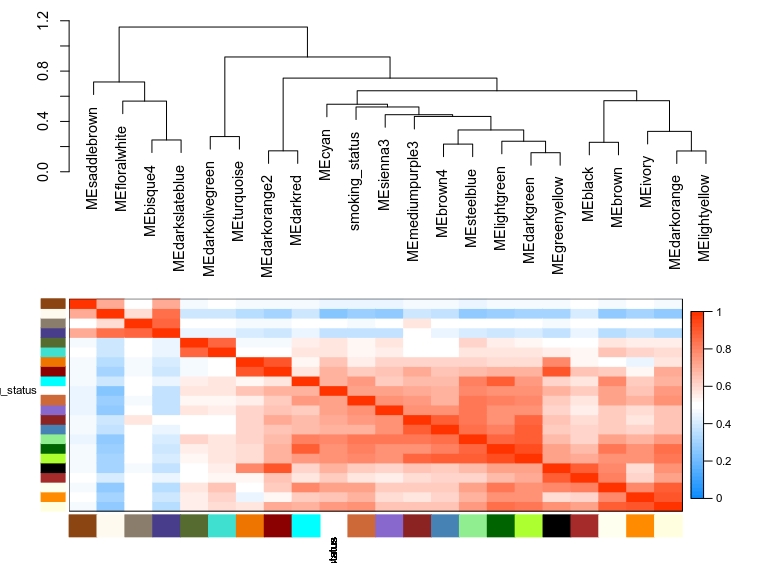
**

**
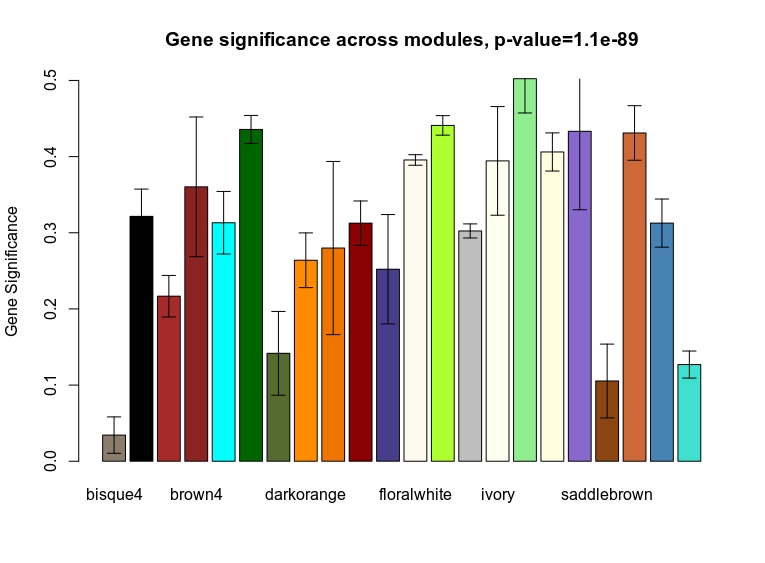
**

**C**

**Supplementary Figure 9 A)** Module-trait associations. Each row corresponds to a module eigengene, column to a trait. Each cell contains the corresponding correlation and p-value. The table is color-coded by correlation according to the color legend. **B)** The eigengene dendrogram and heatmap identify groups of correlated eigengenes. **C)**Barplot of mean gene significance across module

| **Cellular Components** |  |  |
| --- | --- | --- |
| **NAME** | **NOM p-val** | **FDR q-val** |
| GO_MICROTUBULE | 0.010 | 0.183 |
| GO_MICROTUBULE_CYTOSKELETON | 0.038 | 0.229 |
| GO_SUPRAMOLECULAR_FIBER | 0.028 | 0.155 |
| GO_SPINDLE | 0.020 | 0.171 |
| GO_MICROTUBULE_ASSOCIATED_COMPLEX | 0.002 | 0.143 |
| GO_CHROMOSOME | 0.024 | 0.145 |
| GO_CYTOSKELETAL_PART | 0.062 | 0.141 |
| GO_MICROTUBULE_ORGANIZING_CENTER | 0.051 | 0.128 |
| GO_CENTROSOME | 0.044 | 0.140 |
| GO_MIDBODY | 0.014 | 0.132 |
| GO_SPINDLE_MICROTUBULE | 0.000 | 0.130 |
| GO_CHROMOSOMAL_REGION | 0.018 | 0.136 |
| GO_NUCLEOLUS | 0.024 | 0.130 |
| GO_CONDENSED_CHROMOSOME | 0.014 | 0.140 |
| GO_MITOTIC_SPINDLE | 0.018 | 0.137 |
| GO_SPINDLE_POLE | 0.034 | 0.132 |
| GO_CHROMOSOME_CENTROMERIC_REGION | 0.032 | 0.135 |
| GO_CONDENSED_NUCLEAR_CHROMOSOME | 0.000 | 0.139 |
| GO_NUCLEAR_CHROMOSOME | 0.057 | 0.141 |
| GO_CHROMATIN | 0.070 | 0.135 |
| GO_KINETOCHORE | 0.048 | 0.138 |
| GO_CONDENSED_CHROMOSOME_OUTER_KINETOCHORE | 0.004 | 0.136 |
| GO_KINESIN_COMPLEX | 0.002 | 0.137 |
| GO_CONDENSED_CHROMOSOME_CENTROMERIC_REGION | 0.026 | 0.134 |
| GO_MICROTUBULE_ORGANIZING_CENTER_PART | 0.054 | 0.141 |
| GO_CYTOSKELETON | 0.115 | 0.143 |
| GO_CONDENSED_NUCLEAR_CHROMOSOME_CENTROMERIC_REGION | 0.002 | 0.142 |
| GO_REPLICATION_FORK | 0.053 | 0.151 |
| GO_SPINDLE_MIDZONE | 0.018 | 0.150 |
| GO_CENTRIOLE | 0.039 | 0.156 |
| GO_UBIQUITIN_LIGASE_COMPLEX | 0.068 | 0.156 |
| GO_PROTEIN_DNA_COMPLEX | 0.053 | 0.158 |
| GO_CHROMOSOME_TELOMERIC_REGION | 0.026 | 0.157 |
| GO_MICROTUBULE_END | 0.000 | 0.170 |
| GO_NUCLEAR_UBIQUITIN_LIGASE_COMPLEX | 0.072 | 0.182 |
| GO_NUCLEAR_CHROMOSOME_TELOMERIC_REGION | 0.038 | 0.206 |
| GO_DNA_PACKAGING_COMPLEX | 0.029 | 0.219 |
| GO_RIBONUCLEOPROTEIN_COMPLEX | 0.101 | 0.215 |
| GO_SPLICEOSOMAL_COMPLEX | 0.095 | 0.219 |
| GO_CULLIN_RING_UBIQUITIN_LIGASE_COMPLEX | 0.105 | 0.217 |
| GO_HETEROCHROMATIN | 0.076 | 0.224 |
| GO_NUCLEAR_PERIPHERY | 0.146 | 0.230 |
| GO_NUCLEAR_REPLICATION_FORK | 0.100 | 0.240 |
| GO_INTERCELLULAR_BRIDGE | 0.062 | 0.236 |

**Supplementary Table 2** List of significant gene sets in cellular components from GSEA analysis.

| **Cannonical Pathways** |  |  |
| --- | --- | --- |
| **NAME** | **NOM p-val** | **FDR q-val** |
| KEGG_SPLICEOSOME | 0.05 | 0.25 |
| KEGG_PYRIMIDINE_METABOLISM | 0.08 | 0.24 |
| REACTOME_MITOTIC_G1_G1_S_PHASES | 0.05 | 0.24 |
| BIOCARTA_G2_PATHWAY | 0.03 | 0.24 |
| PID_P73PATHWAY | 0.04 | 0.24 |
| REACTOME_ACTIVATION_OF_ATR_IN_RESPONSE_TO_REPLICATION_STRESS | 0.04 | 0.24 |
| KEGG_OOCYTE_MEIOSIS | 0.11 | 0.23 |
| PID_ATR_PATHWAY | 0.06 | 0.23 |
| REACTOME_M_G1_TRANSITION | 0.06 | 0.22 |
| REACTOME_ACTIVATION_OF_THE_PRE_REPLICATIVE_COMPLEX | 0.05 | 0.21 |
| REACTOME_METABOLISM_OF_NUCLEOTIDES | 0.06 | 0.22 |
| PID_FANCONI_PATHWAY | 0.06 | 0.22 |
| KEGG_PROGESTERONE_MEDIATED_OOCYTE_MATURATION | 0.12 | 0.22 |
| REACTOME_E2F_MEDIATED_REGULATION_OF_DNA_REPLICATION | 0.08 | 0.21 |
| REACTOME_DNA_STRAND_ELONGATION | 0.09 | 0.21 |
| REACTOME_TELOMERE_MAINTENANCE | 0.07 | 0.22 |
| REACTOME_RECRUITMENT_OF_MITOTIC_CENTROSOME_PROTEINS_AND_COMPLEXES | 0.09 | 0.22 |
| KEGG_P53_SIGNALING_PATHWAY | 0.14 | 0.21 |
| KEGG_HOMOLOGOUS_RECOMBINATION | 0.04 | 0.21 |
| REACTOME_APC_CDC20_MEDIATED_DEGRADATION_OF_NEK2A | 0.03 | 0.21 |
| KEGG_DNA_REPLICATION | 0.07 | 0.20 |
| PID_AURORA_A_PATHWAY | 0.07 | 0.20 |
| REACTOME_EXTENSION_OF_TELOMERES | 0.09 | 0.21 |
| PID_ATM_PATHWAY | 0.05 | 0.20 |
| REACTOME_REGULATION_OF_MITOTIC_CELL_CYCLE | 0.15 | 0.20 |
| REACTOME_LOSS_OF_NLP_FROM_MITOTIC_CENTROSOMES | 0.11 | 0.20 |
| REACTOME_ORC1_REMOVAL_FROM_CHROMATIN | 0.04 | 0.21 |
| REACTOME_ASSEMBLY_OF_THE_PRE_REPLICATIVE_COMPLEX | 0.05 | 0.21 |
| REACTOME_FACTORS_INVOLVED_IN_MEGAKARYOCYTE_DEVELOPMENT_AND_PLATELET_PRODUCTION | 0.14 | 0.21 |
| KEGG_NUCLEOTIDE_EXCISION_REPAIR | 0.10 | 0.21 |
| REACTOME_TRANSCRIPTION_COUPLED_NER_TC_NER | 0.10 | 0.20 |
| REACTOME_NUCLEOTIDE_EXCISION_REPAIR | 0.10 | 0.20 |
| REACTOME_REPAIR_SYNTHESIS_FOR_GAP_FILLING_BY_DNA_POL_IN_TC_NER | 0.10 | 0.20 |
| REACTOME_GLOBAL_GENOMIC_NER_GG_NER | 0.10 | 0.19 |
| REACTOME_G1_S_SPECIFIC_TRANSCRIPTION | 0.11 | 0.19 |
| REACTOME_DEPOSITION_OF_NEW_CENPA_CONTAINING_NUCLEOSOMES_AT_THE_CENTROMERE | 0.12 | 0.20 |
| REACTOME_APC_C_CDC20_MEDIATED_DEGRADATION_OF_MITOTIC_PROTEINS | 0.12 | 0.20 |
| REACTOME_G0_AND_EARLY_G1 | 0.09 | 0.20 |
| REACTOME_MEIOTIC_RECOMBINATION | 0.13 | 0.20 |
| KEGG_BASE_EXCISION_REPAIR | 0.10 | 0.20 |
| PID_E2F_PATHWAY | 0.17 | 0.20 |
| KEGG_MISMATCH_REPAIR | 0.12 | 0.23 |
| REACTOME_LAGGING_STRAND_SYNTHESIS | 0.12 | 0.23 |
| REACTOME_UNWINDING_OF_DNA | 0.15 | 0.23 |
| REACTOME_MEIOSIS | 0.20 | 0.25 |
| REACTOME_APC_C_CDH1_MEDIATED_DEGRADATION_OF_CDC20_AND_OTHER_APC_C_CDH1_TARGETED_PROTEINS_IN_LATE_MITOSIS_EARLY_G1 | 0.17 | 0.25 |

**Supplementary Table 3** List of significant gene sets in canonical pathways from GSEA analysis.

| **Transcription Factor Targets** |  |  |
| --- | --- | --- |
| **NAME** | **NOM p-val** | **FDR q-val** |
| V$E2F1_Q6 | 0.021 | 0.237 |
| V$E2F_Q4 | 0.018 | 0.206 |
| V$E2F_Q6 | 0.018 | 0.181 |
| SGCGSSAAA_V$E2F1DP2_01 | 0.021 | 0.218 |
| V$E2F1DP1RB_01 | 0.031 | 0.210 |
| V$E2F_Q3_01 | 0.042 | 0.245 |
| V$E2F1_Q3 | 0.057 | 0.240 |

**Supplementary Table 4** List of significant gene sets in transcription factor targets from GSEA analysis.


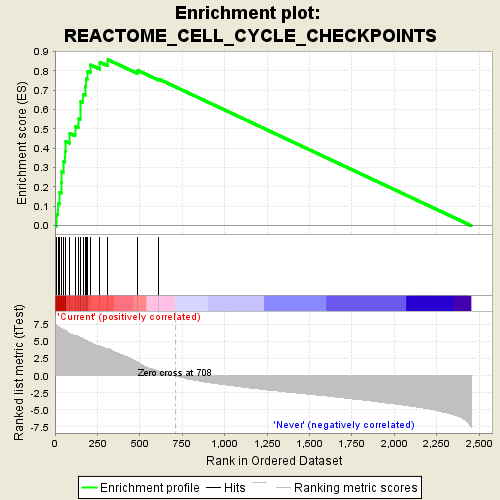

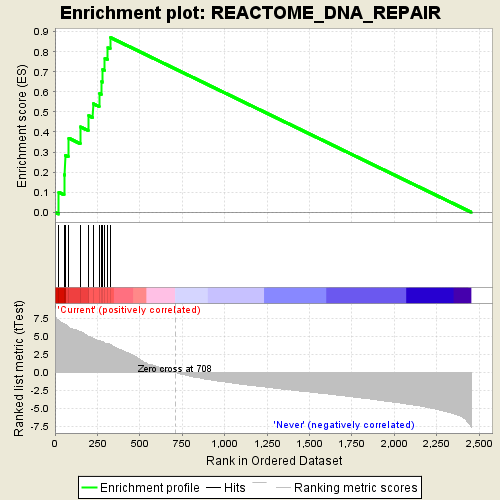

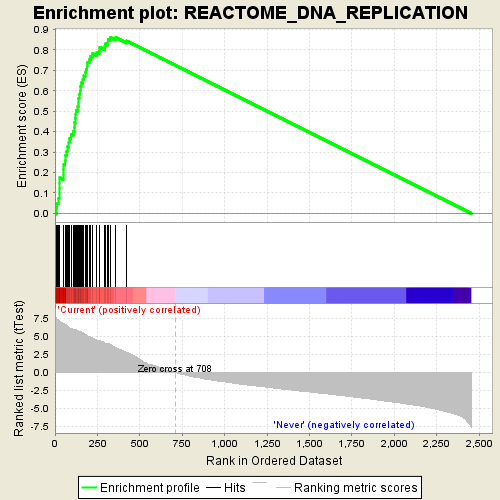

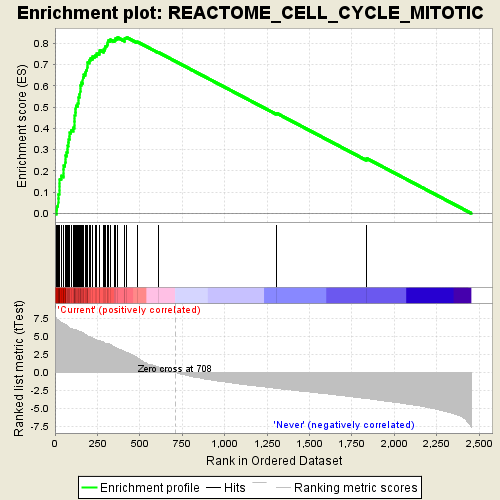

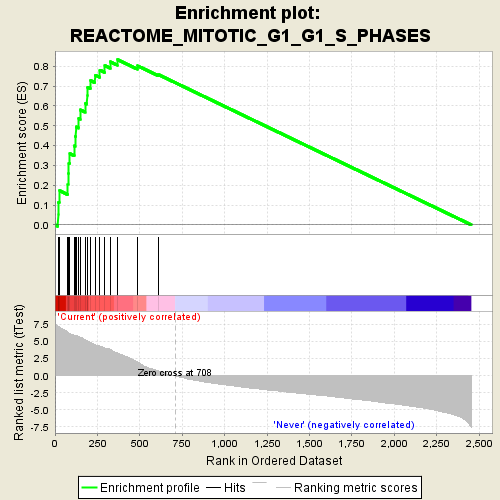

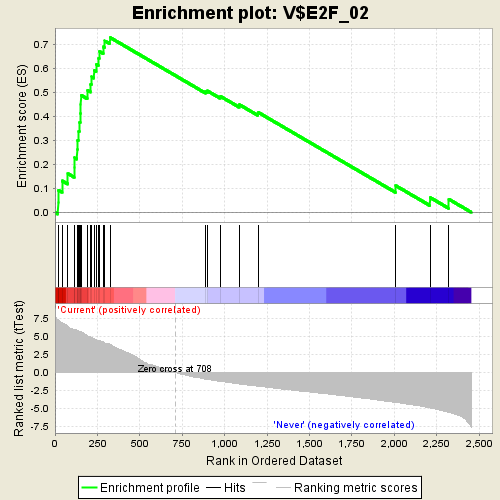


**p <0.06**

**FDR<0.031**

**p <0.008**

**FDR=0.114**

**p <0.01**

**FDR=0.190**

**p <0.01**

**FDR=0.16**

**p <0.004**

**FDR=0.109**

**p <0.08**

**FDR=0.113**

**Supplementary Figure 10.** Enrichment plots showing 2454 genes to be involved in cell cycle, DNA repair, replication and E2F family of transcription factors.

| **Pathway or Process** | **XD-score** | **q-value** | **Overlap/Size** |
| --- | --- | --- | --- |
| *Drug metabolism - other enzymes* | 0.24236 | 1 | 1/16 |
| *Glycine, serine and threonine metabolism* | 0.21458 | 1 | 1/18 |
| ***ABC transporters*** | 0.19236 | 1 | 1/20 |
| ***Mismatch repair*** | 0.16627 | 1 | 1/23 |
| *Glutathione metabolism* | 0.15047 | 1 | 1/26 |
| *Cysteine and methionine metabolism* | 0.1405 | 1 | 1/27 |
| *Arrhythmogenic right ventricular cardiomyopathy (ARVC)* | 0.12139 | 1 | 2/62 |
| ***Cell cycle*** | 0.09236 | 1 | 3/120 |
| *Colorectal cancer* | 0.05793 | 1 | 1/61 |
| *Pyrimidine metabolism* | 0.0495 | 1 | 1/70 |
| *Progesterone-mediated oocyte maturation* | 0.04299 | 1 | 1/79 |
| *Parkinson's disease* | 0.03276 | 1 | 1/99 |
| *Ubiquitin mediated proteolysis* | 0.02597 | 1 | 1/119 |
| *Metabolic pathways* | 0.01753 | 1 | 4/640 |
| *Pathways in cancer* | 0.00551 | 1 | 1/304 |
| ***Non-homologous end-joining*** | 0.00090 | 1 | 0/13 |

**Supplementary Table 5.** Jepetto ontology analysis.

| **GENE** | **Average Current Smokers (CS)** | **Average Non-Smokers (NS)** | **CS/NS** | **Log_2_FC** |
| --- | --- | --- | --- | --- |
| COL10A1 | 1103.663 | 2191.056 | 0.504 | -0.989 |
| CDK14 | 342.927 | 549.827 | 0.624 | -0.681 |
| HELLS | 193.926 | 96.863 | 2.002 | 1.001 |
| ATAD2 | 1473.042 | 733.996 | 2.007 | 1.005 |
| MCM2 | 1703.348 | 846.390 | 2.012 | 1.009 |
| PSRC1 | 221.796 | 110.146 | 2.014 | 1.010 |
| SLC7A1 | 1458.927 | 723.354 | 2.017 | 1.012 |
| WDR67 | 244.609 | 121.246 | 2.017 | 1.013 |
| IQGAP3 | 813.292 | 402.823 | 2.019 | 1.014 |
| PCDH10 | 35.514 | 17.567 | 2.022 | 1.015 |
| GTSE1 | 306.295 | 151.115 | 2.027 | 1.019 |
| MAPK8IP2 | 265.397 | 130.907 | 2.027 | 1.020 |
| C9orf100 | 223.191 | 109.770 | 2.033 | 1.024 |
| ERCC6L | 150.463 | 73.835 | 2.038 | 1.027 |
| C15orf23 | 452.914 | 221.775 | 2.042 | 1.030 |
| ECT2 | 1578.469 | 762.100 | 2.071 | 1.050 |
| HIST1H2BJ | 59.507 | 28.638 | 2.078 | 1.055 |
| CDC6 | 674.595 | 324.381 | 2.080 | 1.056 |
| KPNA2 | 3351.393 | 1609.287 | 2.083 | 1.058 |
| POLE2 | 143.312 | 68.531 | 2.091 | 1.064 |
| FBXO43 | 23.434 | 11.154 | 2.101 | 1.071 |
| GPR19 | 31.429 | 14.950 | 2.102 | 1.072 |
| MAD2L1 | 553.410 | 263.098 | 2.103 | 1.073 |
| PGC | 39692.112 | 18856.802 | 2.105 | 1.074 |
| LOC730101 | 218.104 | 103.570 | 2.106 | 1.074 |
| E2F8 | 237.507 | 112.652 | 2.108 | 1.076 |
| CENPW | 212.788 | 100.871 | 2.110 | 1.077 |
| MTBP | 145.517 | 68.953 | 2.110 | 1.077 |
| E2F1 | 692.107 | 327.445 | 2.114 | 1.080 |
| ZWINT | 951.809 | 449.995 | 2.115 | 1.081 |
| C17orf53 | 172.222 | 80.714 | 2.134 | 1.093 |
| XRCC2 | 105.523 | 49.330 | 2.139 | 1.097 |
| C16orf59 | 183.487 | 85.697 | 2.141 | 1.098 |
| PFN2 | 3116.626 | 1453.851 | 2.144 | 1.100 |
| CHML | 1280.759 | 596.391 | 2.148 | 1.103 |
| TOP2A | 3852.883 | 1791.266 | 2.151 | 1.105 |
| FANCI | 1017.908 | 472.786 | 2.153 | 1.106 |
| CCNB1 | 1245.186 | 577.687 | 2.155 | 1.108 |
| CCNB1 | 1245.186 | 577.687 | 2.155 | 1.108 |
| EME1 | 112.635 | 52.042 | 2.164 | 1.114 |
| FAM24B | 56.182 | 25.693 | 2.187 | 1.129 |
| C12orf48 | 217.745 | 99.474 | 2.189 | 1.130 |
| TET1 | 97.011 | 44.276 | 2.191 | 1.132 |
| CDKN3 | 244.505 | 111.355 | 2.196 | 1.135 |
| CDK1 | 1082.311 | 492.400 | 2.198 | 1.136 |
| PLK4 | 257.557 | 116.994 | 2.201 | 1.138 |
| AURKAPS1 | 22.849 | 10.372 | 2.203 | 1.139 |
| CENPI | 85.598 | 38.850 | 2.203 | 1.140 |
| E2F2 | 236.343 | 107.056 | 2.208 | 1.143 |
| TRIP13 | 675.124 | 305.692 | 2.209 | 1.143 |
| NUSAP1 | 924.979 | 418.549 | 2.210 | 1.144 |
| MEX3A | 1001.240 | 451.238 | 2.219 | 1.150 |
| GINS2 | 403.024 | 180.838 | 2.229 | 1.156 |
| UHRF1 | 487.746 | 218.353 | 2.234 | 1.159 |
| C11orf82 | 205.145 | 91.557 | 2.241 | 1.164 |
| RACGAP1 | 1135.381 | 503.190 | 2.256 | 1.174 |
| WDR62 | 332.705 | 147.354 | 2.258 | 1.175 |
| FAM83D | 491.591 | 217.409 | 2.261 | 1.177 |
| KIAA0101 | 522.501 | 230.944 | 2.262 | 1.178 |
| CDC25C | 120.725 | 53.125 | 2.272 | 1.184 |
| DEPDC1B | 257.418 | 112.850 | 2.281 | 1.190 |
| CBS | 1126.467 | 492.274 | 2.288 | 1.194 |
| TUBB4 | 212.405 | 92.572 | 2.294 | 1.198 |
| CLSPN | 217.245 | 93.686 | 2.319 | 1.213 |
| CCNA2 | 647.960 | 277.940 | 2.331 | 1.221 |
| DNA2 | 242.797 | 103.937 | 2.336 | 1.224 |
| CHEK1 | 358.058 | 153.272 | 2.336 | 1.224 |
| GSG2 | 83.272 | 35.308 | 2.358 | 1.238 |
| MAP6D1 | 73.981 | 31.246 | 2.368 | 1.244 |
| FAM54A | 121.237 | 51.137 | 2.371 | 1.245 |
| KIF4A | 636.338 | 265.237 | 2.399 | 1.263 |
| C18orf56 | 23.195 | 9.652 | 2.403 | 1.265 |
| KIAA1524 | 263.467 | 109.623 | 2.403 | 1.265 |
| RAD51 | 204.059 | 84.683 | 2.410 | 1.269 |
| RAD51AP1 | 313.278 | 129.814 | 2.413 | 1.271 |
| SPC25 | 146.814 | 60.645 | 2.421 | 1.276 |
| MKI67 | 2461.910 | 1014.638 | 2.426 | 1.279 |
| PRR11 | 160.239 | 65.968 | 2.429 | 1.280 |
| CASC5 | 210.043 | 85.991 | 2.443 | 1.288 |
| CENPF | 1758.249 | 718.891 | 2.446 | 1.290 |
| RAD54L | 238.043 | 96.379 | 2.470 | 1.304 |
| SPAG5 | 1046.252 | 422.935 | 2.474 | 1.307 |
| NEK2 | 523.566 | 211.016 | 2.481 | 1.311 |
| DEPDC1 | 396.547 | 159.790 | 2.482 | 1.311 |
| ASPM | 657.135 | 264.757 | 2.482 | 1.312 |
| RRM2 | 1789.905 | 720.811 | 2.483 | 1.312 |
| CDCA8 | 659.651 | 265.366 | 2.486 | 1.314 |
| CENPE | 367.780 | 147.839 | 2.488 | 1.315 |
| PLK1 | 791.228 | 315.623 | 2.507 | 1.326 |
| PLK1 | 791.228 | 315.623 | 2.507 | 1.326 |
| PKMYT1 | 369.793 | 147.319 | 2.510 | 1.328 |
| DLGAP5 | 496.263 | 197.427 | 2.514 | 1.330 |
| MELK | 464.370 | 184.240 | 2.520 | 1.334 |
| C1orf135 | 85.465 | 33.848 | 2.525 | 1.336 |
| STC2 | 620.176 | 245.168 | 2.530 | 1.339 |
| CDT1 | 534.413 | 210.613 | 2.537 | 1.343 |
| DSCC1 | 250.296 | 98.152 | 2.550 | 1.351 |
| KIF11 | 830.158 | 325.337 | 2.552 | 1.351 |
| BUB1 | 739.387 | 287.960 | 2.568 | 1.360 |
| ORC1L | 235.641 | 91.397 | 2.578 | 1.366 |
| EIF4EBP1 | 1452.756 | 562.420 | 2.583 | 1.369 |
| CDCA2 | 170.324 | 65.480 | 2.601 | 1.379 |
| ADAM11 | 15.649 | 6.013 | 2.603 | 1.380 |
| HMGA1 | 7013.606 | 2689.729 | 2.608 | 1.383 |
| LOC399815 | 27.017 | 10.339 | 2.613 | 1.386 |
| CEP55 | 681.463 | 260.121 | 2.620 | 1.389 |
| CCNB2 | 640.193 | 244.164 | 2.622 | 1.391 |
| SGOL1 | 112.840 | 42.941 | 2.628 | 1.394 |
| MAST1 | 65.036 | 24.620 | 2.642 | 1.401 |
| GGH | 1079.431 | 407.746 | 2.647 | 1.405 |
| BLM | 260.030 | 98.067 | 2.652 | 1.407 |
| RECQL4 | 683.345 | 257.519 | 2.654 | 1.408 |
| PRC1 | 1163.095 | 437.973 | 2.656 | 1.409 |
| SKA3 | 214.751 | 80.470 | 2.669 | 1.416 |
| SKA3 | 214.751 | 80.470 | 2.669 | 1.416 |
| KIF14 | 298.057 | 111.261 | 2.679 | 1.422 |
| CDC45 | 325.860 | 120.739 | 2.699 | 1.432 |
| HJURP | 521.504 | 192.942 | 2.703 | 1.435 |
| POLQ | 172.334 | 63.746 | 2.703 | 1.435 |
| MFSD2B | 88.541 | 32.682 | 2.709 | 1.438 |
| KIF15 | 248.281 | 91.605 | 2.710 | 1.438 |
| NUF2 | 420.141 | 154.975 | 2.711 | 1.439 |
| CKAP2L | 269.989 | 98.952 | 2.728 | 1.448 |
| NDC80 | 392.228 | 143.071 | 2.742 | 1.455 |
| CAMK2N2 | 55.258 | 20.119 | 2.747 | 1.458 |
| ARHGAP11A | 594.344 | 216.354 | 2.747 | 1.458 |
| KIF23 | 586.484 | 212.252 | 2.763 | 1.466 |
| ZYG11A | 85.760 | 31.019 | 2.765 | 1.467 |
| NEIL3 | 119.447 | 43.145 | 2.769 | 1.469 |
| NCAPG | 611.439 | 220.837 | 2.769 | 1.469 |
| NCAPG | 611.439 | 220.837 | 2.769 | 1.469 |
| CDC20 | 1146.755 | 414.059 | 2.770 | 1.470 |
| CDC20 | 1146.755 | 414.059 | 2.770 | 1.470 |
| CDCA3 | 308.280 | 109.917 | 2.805 | 1.488 |
| OIP5 | 118.100 | 42.034 | 2.810 | 1.490 |
| UBE2S | 657.564 | 233.387 | 2.817 | 1.494 |
| PBK | 332.972 | 117.812 | 2.826 | 1.499 |
| UCK2 | 493.989 | 174.641 | 2.829 | 1.500 |
| E2F7 | 145.859 | 51.429 | 2.836 | 1.504 |
| CDCA5 | 778.608 | 273.824 | 2.843 | 1.508 |
| PIF1 | 101.463 | 35.658 | 2.845 | 1.509 |
| TTK | 340.658 | 119.407 | 2.853 | 1.512 |
| SPC24 | 36.380 | 12.683 | 2.868 | 1.520 |
| BIRC5 | 622.265 | 216.678 | 2.872 | 1.522 |
| CCDC150 | 57.169 | 19.863 | 2.878 | 1.525 |
| SLC7A5 | 4660.863 | 1618.214 | 2.880 | 1.526 |
| CDC25A | 180.257 | 62.489 | 2.885 | 1.528 |
| BUB1B | 531.601 | 183.359 | 2.899 | 1.536 |
| BUB1B | 531.601 | 183.359 | 2.899 | 1.536 |
| PLEKHH1 | 285.342 | 98.084 | 2.909 | 1.541 |
| KIFC1 | 859.561 | 293.888 | 2.925 | 1.548 |
| NCAPH | 457.413 | 155.389 | 2.944 | 1.558 |
| AURKB | 412.571 | 138.189 | 2.986 | 1.578 |
| FOXM1 | 1449.568 | 482.988 | 3.001 | 1.586 |
| KIF18B | 415.398 | 138.329 | 3.003 | 1.586 |
| EXO1 | 320.884 | 106.420 | 3.015 | 1.592 |
| C9orf140 | 684.997 | 224.764 | 3.048 | 1.608 |
| CBX2 | 616.301 | 202.082 | 3.050 | 1.609 |
| KIF2C | 795.619 | 259.905 | 3.061 | 1.614 |
| DSP | 12182.704 | 3975.292 | 3.065 | 1.616 |
| DSCR6 | 72.575 | 23.677 | 3.065 | 1.616 |
| DNMT3B | 198.636 | 63.791 | 3.114 | 1.639 |
| PKP2 | 534.496 | 171.534 | 3.116 | 1.640 |
| TPX2 | 1859.743 | 593.527 | 3.133 | 1.648 |
| TROAP | 437.026 | 139.418 | 3.135 | 1.648 |
| C15orf42 | 188.053 | 59.945 | 3.137 | 1.649 |
| FAM64A | 219.893 | 70.000 | 3.141 | 1.651 |
| CENPA | 221.829 | 70.220 | 3.159 | 1.660 |
| FAM72A | 45.560 | 14.352 | 3.174 | 1.667 |
| FAM72B | 249.534 | 78.487 | 3.179 | 1.669 |
| MYBL2 | 1746.090 | 545.309 | 3.202 | 1.679 |
| ESPL1 | 479.663 | 146.043 | 3.284 | 1.716 |
| UBE2C | 1114.089 | 338.837 | 3.288 | 1.717 |
| RNFT2 | 181.622 | 54.341 | 3.342 | 1.741 |
| FAM72D | 143.209 | 42.175 | 3.396 | 1.764 |
| CTSL2 | 272.117 | 79.776 | 3.411 | 1.770 |
| MCM10 | 300.883 | 86.715 | 3.470 | 1.795 |
| CYP4F22 | 18.884 | 5.398 | 3.498 | 1.807 |
| LASS1 | 26.440 | 7.557 | 3.499 | 1.807 |
| TXNRD1 | 12460.911 | 3502.463 | 3.558 | 1.831 |
| SKA1 | 208.091 | 57.316 | 3.631 | 1.860 |
| CABP7 | 46.322 | 12.687 | 3.651 | 1.868 |
| UCHL1 | 2764.699 | 719.110 | 3.845 | 1.943 |
| ASXL3 | 72.656 | 18.605 | 3.905 | 1.965 |
| HES6 | 1302.454 | 314.761 | 4.138 | 2.049 |
| GPC2 | 88.922 | 21.271 | 4.181 | 2.064 |
| GCLC | 3866.484 | 922.099 | 4.193 | 2.068 |
| LRRC16B | 59.894 | 14.186 | 4.222 | 2.078 |
| MLLT11 | 652.937 | 147.855 | 4.416 | 2.143 |
| GLDC | 230.230 | 50.770 | 4.535 | 2.181 |
| HPDL | 80.714 | 15.912 | 5.073 | 2.343 |
| MTL5 | 171.734 | 33.037 | 5.198 | 2.378 |
| PRH2 | 34.155 | 5.375 | 6.354 | 2.668 |
| ABCC2 | 645.006 | 43.092 | 14.968 | 3.904 |

**Supplementary Table 6.** DEGs of relevance to our discussion.

| **Kruskal Wallis p-value=0.0378** |  |  |  |  |
| --- | --- | --- | --- | --- |
| **HEY2** | **FN** | **FC** | **MN** | **MC** |
| **FN** | - | p=0.048 | - | - |
| **FC** | p=0.048 | - | - | - |
| **MN** | - | - | - | - |
| **MC** | - | - | - | - |
|  |  |  |  |  |
|  |  |  |  |  |
| **Kruskal Wallis p-value<0.05** |  |  |  |  |
| **OLFM1** | **FN** | **FC** | **MN** | **MC** |
| **FN** | - | p<0.05 | - | p<0.05 |
| **FC** | p<0.05 | - | - | - |
| **MN** | - | - | - | - |
| **MC** | p<0.05 | - | - | - |
|  |  |  |  |  |
|  |  |  |  |  |
| **Kruskal Wallis p-value=0.0109** |  |  |  |  |
| **SFRP1** | **FN** | **FC** | **MN** | **MC** |
| **FN** | - | - | - | - |
| **FC** | - | - | - | - |
| **MN** | - | - | - | p=0.0391 |
| **MC** | - | - | p=0.0391 | - |
|  |  |  |  |  |
|  |  |  |  |  |
| **Kruskal Wallis p-value=0.0001** |  |  |  |  |
| **STRAP** | **FN** | **FC** | **MN** | **MC** |
| **FN** | - | p=0.0031 | - | p=0.0001 |
| **FC** | p=0.0031 | - | - | - |
| **MN** | - | - | - | - |
| **MC** | p=0.0001 | - | - | - |

**Supplementary Table 7.** Statistical analysis for the statistically significant EMT marker genes for FN, FC, MN, and MC groups.

| **Gene** | **Log_2_FC change NS** |  | **Gene** | **Log_2_FC change CS** |
| --- | --- | --- | --- | --- |
| **SYT1** | -2.28 |  | **WIF1** | -4.88 |
| **UCHL1** | -1.74 |  | **ASXL3** | -3.77 |
| **AOX1** | -1.74 |  | **CYP4F22** | -3.65 |
| **MLLT11** | -1.65 |  | **CLDN18** | -3.14 |
| **GINS4** | -1.61 |  | **PRH2** | -3.09 |
| **CNTNAP2** | -1.58 |  | **LRRC36** | -1.87 |
| **C10orf105** | 1.53 |  | **ZNF804A** | -1.82 |
| **FCER2** | 1.54 |  | **KLKB1** | -1.53 |
| **FAM95B1** | 1.55 |  | **PRG4** | -1.50 |
| **CTSH** | 1.60 |  | **REEP1** | 1.54 |
| **GPD1** | 1.88 |  | **PLA2G2A** | 4.65 |
| **OGN** | 2.13 |  |  |  |
| **ALDH1A2** | 2.32 |  |  |  |
| **PLA2G2A** | 3.19 |  |  |  |
| **PRG4** | 3.77 |  |  |  |

**Supplementary Table 8.** Log fold change between females and males with1.5 <log_2_FC < -1.5.

| **Cell Cycle** | *CD22*, *BTK*, ***PIK3R5*** (NG_030374.1), ***PIK3CG*** (NG_050579.1), *CD19* (NG_007275.1)*, PRKCB* (NG_029003.2)*, INPP5D* (NG_033988.1)*, NFATC2,PIK3R1* (NG_012849.2)*,PIK3AP1,FCGR2B*(NG_023318.1)*,RAC2* (NG_007288.1) |
| --- | --- |
| **Mismatch Repair** | ***POLD1*** (NG_033800.1)*,* ***RFC5, RFC4, RFC3,*** *EXO1* (NG_029100.2) |
| **Homologous Recombination** | ***POLD1*** (NG_033800.1), *RAD54B* (NG_012878.2), *XRCC2* (NG_027988.2), *EME1* (NG_029665.1), *RAD51* (NG_012120.1), *RAD54L* (NG_012144.1), *BLM* (NG_007272.1) |
| **DNA Replication** | *DNA2* (NG_034247.1)*, MCM2* (NG_050771.1)*,* ***POLD1***(NG_033800.1)*,POLE2* (NG_052877.1)*, POLE* (NG_033840.1)*,* ***RFC3, RFC4, RFC5*** |
| **VEGF signalling pathway** | ***PIK3R5*** (NG_030374.1)***, PLA2G2A***(NG_012928.1),  ***PIK3CG*** (NG_050579.1), ***PRKCB*** (NG_029003.2), ***NFATC2***  ***PLA2G5*** (NG_032045.1), ***PIK3R1*** (NG_012849.2),  ***PLA2G2D, RAC2*** (NG_007288.1) |
| **T cell receptor signaling pathway** | ***CD40LG***(NG_007280.1), *CSF2* (NG_033024.1), *CD28* (NG_029618.1), *PTPRC* (NG_007730.1), ***PIK3R5*** (NG_030374.1), *CD4* (NG_027688.1), *ITK* (NG_016276.1)  ***PIK3CG*** (NG_050579.1)***, NFATC2, PIK3R1*** (NG_012849.2) |
| **ErbB signaling pathway** | ***PIK3R5*** (NG_030374.1),***PIK3CG*** (NG_050579.1), ***MAPK10*** (NG_013325.2), ***PRKCB*** (NG_029003.2), ***PIK3R1*** (NG_012849.2), *BTC* |
| **GnRH signaling pathway** | ***PLA2G2A*** (NG_012928.1), ***MAPK10*** (NG_013325.2),  *PLCB2* (NG_052867.1), *CACNA1C* (NG_008801.2), ***PRKCB***(NG_029003.2), *PTK2B* (NG_029510.2), ***PLA2G5*** (NG_032045.1), ***PLA2G2D*** |
| **Base excision repair** | *TDG* |
| **Asthma** | ***CD40LG*** (NG_007280.1),*FCER1A, FCER1G, HLA-DMA, HLA-DMB, HLA-DOA, HLA-DOB, HLA-DPA1, HLA-DPB1, HLA-DQA1, HLA-DQB1, HLA-DRA, HLA-DRB1, HLA-DRB5, MS4A2* |

**Supplementary Table 9.** Genes involved in major cancer related pathways coloured according to increased expression in males in red, and females for blue

| **CbioPortal Lung Adenocarcinoma (TCGA, Provisional)** | | | | | | |
| --- | --- | --- | --- | --- | --- | --- |
| Subgroup | **C1** | **C2** | **N1** | **N2** | p-value | additional test |
| Gene | Samples with mutations | Samples with mutations | Samples with mutations | Samples with mutations |  |  |
| MUC17 | 5 | 7 | 1 | 1 | X-squared = 3.4238, df = 1, p-value = 0.06426 |  |
| SPTA1 | 5 | 4 | 2 | 1 | 0.5357 | Fisher's Exact Test for Count Data |
| **LRP1B** | 6 | 9 | 1 |  | X-squared = 7.2838, df = 1, p-value = 0.006958 |  |
| **ZFHX4** | 6 | 9 | 1 |  | X-squared = 7.2838, df = 1, p-value = 0.006958 |  |
| KRAS | 7 | 7 | 2 | 2 | X-squared = 1.9202, df = 1, p-value = 0.1658 |  |
| **EGFR** | 1 | 1 | 6 | 3 | p-value = 0.002245 | Fisher's Exact Test for Count Data |
| FLG | 9 | 6 | 3 | 1 | X-squared = 2.3794, df = 1, p-value = 0.1229 |  |
| **MUC16** | 10 | 8 |  | 2 | X-squared = 7.2414, df = 1, p-value = 0.007124 |  |
| **RYR2** | 12 | 10 | 5 |  | X-squared = 4.8476, df = 1, p-value = 0.02769 |  |
| **TP53** | 13 | 10 | 4 | 2 | X-squared = 4.1237, df = 1, p-value = 0.04229 |  |
| PIK3CA | 2 | 2 | 1 |  | 0.6516 | Fisher's Exact Test for Count Data |
| USH2A | 6 | 5 | 1 | 2 | X-squared = 1.6081, df = 1, p-value = 0.2048 |  |
| STK11 | 3 | 4 | 1 | 1 | 0.4854 | Fisher's Exact Test for Count Data |
| **NAV3** | 6 | 5 | 1 |  | 0.03284 | Fisher's Exact Test for Count Data |
| RYR3 | 1 | 5 |  | 1 | 0.2601 | Fisher's Exact Test for Count Data |
| KEAP1 | 5 | 3 | 2 |  | 0.3239 | Fisher's Exact Test for Count Data |
| ZFHX3 |  | 2 |  | 2 | 0.6267 | Fisher's Exact Test for Count Data |
| BRINP1 | 2 | 2 | 2 |  | 1 | Fisher's Exact Test for Count Data |
| COL6A3 | 2 | 4 |  | 2 | 0.7104 |  |
| TTN | 9 | 8 | 4 | 3 | X-squared = 0.75353, df = 1, p-value = 0.3854 |  |
| MET | 1 |  |  | 2 | 0.5551 |  |
| ZNF536 | 7 | 2 | 2 |  | X-squared = 1.8049, df = 1, p-value = 0.1791 |  |
| AHNAK2 | 8 | 4 | 2 |  | X-squared = 3.4238, df = 1, p-value = 0.06426 |  |
| AKAP9 | 1 | 1 | 1 |  | 1 |  |
| ANK3 | 1 | 2 | 2 |  | 1 |  |
| CPS1 | 3 | 5 |  | 2 | 0.3239 |  |
| DMD | 3 |  | 2 |  | 1 |  |
| FAT3 | 4 | 3 |  | 3 | 0.7452 |  |
| PCDH15 | 4 | 5 | 1 |  | 0.0918 |  |
| ZNF804A | 4 | 7 |  | 1 | 0.03284 |  |
| DNAH5 | 6 | 2 | 1 | 1 | 0.3239 |  |
| Total mutations | 183 | 179 | 54 | 34 |  |  |

**Supplementary Table10.** Mutation analysis

| **CbioPortal Lung Adenocarcinoma (TCGA, Provisional)** | | | | |
| --- | --- | --- | --- | --- |
| **Gene** | **C1 (22/54)** | **C2 (16/38)** | **C3 (7/28)** | **p value** |
|  | **Freq** | **Freq** | **Freq** |  |
| TTN | 40.91% | 50% | 42.86% | 0.853 |
| RYR2 | 54.55% | 62.50% | 57.14% | 0.886 |
| MUC16 | 45.45% | 50% | 28.57% | 0.630 |
| FLG | 40.91% | 37.50% | 42.86% | 0.964 |
| TP53 | 59.09% | 62.50% | 71.43% | 0.842 |
| XIRP2 | 31.82% | 18.75% | 42.86% | 0.459 |
| AHNAK2 | 36.36% | 25% | 57.14% | 0.332 |
| PCDH15 | 18.18% | 31.25% | 57.14% | 0.136 |
| LRP1B | 27.27% | 56.25% | 57.14% | 0.139 |
| ZNF536 | 31.82% | 12.50% | 28.57% | 0.378 |
| NAV3 | 27.27% | 31.25% | 42.86% | 0.740 |
| DST | 22.73% | 18.75% | 42.86% | 0.449 |
| ZFHX4 | 27.27% | 56.25% | 42.86% | 0.195 |
| USH2A | 27.27% | 31.25% | 28.57% | 0.965 |
| APOB | 13.64% | 31.25% | 42.86% | 0.215 |
| BCLAF1 | 4.55% | 43.75% | 14.29% | 0.0107 |
| SAMD9 | 4.55% | 12.50% | 57.14% | 0.00341 |
| ADAMTS12 | 22.73% | 25% | 42.86% | 0.567 |
| FAT3 | 18.18% | 18.75% | 57.14% | 0.091 |
| SI | 13.64% | 18.75% | 42.86% | 0.239 |
| MUC17 | 22.73% | 43.75% | 42.86% | 0.336 |
| ZNF804A | 18.18% | 43.75% | 28.57% | 0.229 |

**Supplementary Table11.** Comparison of the occurrence of mutations in the three subgroups of the current smoker. Percentage of samples with mutations (Freq) is presented. P-value was calculated by chi square test. Results statistically significant (p <0.05) are highlighted in pink.

| **CbioPortal Lung Adenocarcinoma (TCGA, Provisional)** | | | | |
| --- | --- | --- | --- | --- |
| **Gene** | **N1 (12/17)** | **N2 (12/37)** | **N3 (8/21)** | **p value** |
|  | **Freq** | **Freq** | **Freq** |  |
| EGFR | 50% | 25% | 12.50% | 0.175 |
| TP53 | 33.33% | 16.67% | 25% | 0.641 |
| TTN | 33.33% | 25% | 25% | 0.879 |
| FRG1BP | 16.67% | 8.33% | 12.50% | 0.827 |
| KRAS | 16.67% | 16.67% | 12.50% | 0.961 |
| SPTA1 | 16.67% | 8.33% | 25% | 0.598 |
| SMAD4 | 8.33% | 8.33% | 12.50% | 0.941 |
| ARHGEF12 | 8.33% | 8.33% | 12.50% | 0.941 |
| USH2A | 8.33% | 16.67% | 12.50% | 0.827 |
| NLRC4 | 8.33% | 8.33% | 12.50% | 0.941 |
| MUC17 | 8.33% | 8.33% | 12.50% | 0.941 |

**Supplementary Table12.** Comparison of the occurrence of mutations in the three subgroups of the non-smokers. Percentage of samples with mutations (Freq) is presented. P-value was calculated by chi square test. There are no statistically significant results (p <0.05).

| **Gene** | **Kruskall-Wallis** | **C1C2** | **C1N1** | **C2N1** | **C1N2** | **C2N2** | **N1N2** |
| --- | --- | --- | --- | --- | --- | --- | --- |
| **ACTA2** | 0.0043 | - | 0.0014 | - | - | - | - |
| **CDH11** | 0.0001 | - | 0.0448 | - | 0.0001 | 0.0009 | - |
| **CTNNB1** | 0.0179 | - | - | 0.0070 | - | - | - |
| **DDR2** | 0.0000 | - | - | - | 0.0001 | 0.0000 | - |
| **DSP** | 0.0000 | - | 0.0004 | 0.0276 | 0.0000 | 0.0003 | - |
| **ITGB6** | 0.0000 | - | 0.0044 | 0.0276 | 0.0002 | 0.0050 | - |
| **KRT18** | 0.0000 | - | - | - | 0.0077 | 0.0000 | - |
| **KRT5** | 0.0188 | - | - | - | 0.0097 | - | - |
| **KRT8** | 0.0005 | 0.0108 | - | - | - | 0.0001 | - |
| **LAMA2** | 0.0000 | - | - | 0.0360 | 0.0000 | 0.0000 | 0.0281 |
| **LAMA3** | 0.0268 | - | - | 0.0338 | - | - | 0.0102 |
| **LAMA4** | 0.0234 | - | - | - | 0.0107 | 0.0471 | - |
| **LAMA5** | 0.0000 | - | 0.0001 | 0.0000 | - | - | 0.0000 |
| **MMP2** | 0.0032 | - | - | - | 0.0011 | - | - |
| **MUC1** | 0.0017 | - | - | - | 0.0004 | 0.0478 | - |
| **OCLN** | 0.0010 | - | - | 0.0020 | - | 0.0033 | - |
| **S100A4** | 0.0152 | - | 0.0249 | - | 0.0295 | - | - |
| **SDC1** | 0.0032 | - | 0.0412 | - | 0.0020 | - | - |
| **SMAD2** | 0.0077 | - | - | - | 0.0018 | - | - |
| **TCF3** | 0.0000 | 0.0134 | 0.0024 | 0.0000 | - | 0.0001 | - |
| **TCF4** | 0.0000 | - | - | 0.0193 | 0.0047 | 0.0000 | - |
| **TJP1** | 0.0229 | - | - | - | - | - | 0.0423 |
| **VIM** | 0.0000 | - | 0.0097 | - | 0.0000 | 0.0006 | - |
| **ZEB1** | 0.0000 | - | - | 0.0313 | 0.0000 | 0.0000 | - |
| **ZEB2** | 0.0000 | - | 0.0461 | 0.0047 | 0.0000 | 0.0000 | - |

**Supplementary Table13.** Statistical analysis for the statistically significant EMT marker genes for C1C2, C1N1, C2N1, C1N2, C2N2, N1N2.

| **Gene** | **Kruskall-Wallis** | **C1C2** | **C1N1** | **C2N1** | **C1N2** | **C2N2** | **N1N2** |
| --- | --- | --- | --- | --- | --- | --- | --- |
| **BLM** | 1.43E-10 | - | 0.0005798 | 0.02025 | 3.87E-10 | 3.28E-06 | - |
| **RAD51** | 3.72E-08 | - | - | - | 1.20E-07 | 9.38E-07 | - |

**Supplementary Table14.** Statistical analysis for the statistically significant drug response related genes for C1C2, C1N1, C2N1, C1N2, C2N2, N1N2.
